# Phototemtide A, a Cyclic Lipopeptide Heterologously Expressed from *Photorhabdus temperata* Meg1, Shows Selective Antiprotozoal Activity

**DOI:** 10.1002/cbic.201900665

**Published:** 2020-02-04

**Authors:** Lei Zhao, Tien Duy Vo, Marcel Kaiser, Helge B. Bode

**Affiliations:** ^1^ Molecular Biotechnology Department of Biosciences Goethe University Frankfurt Max-von-Laue-Strasse 9 60438 Frankfurt am Main Germany; ^2^ Institute of Botany Jiangsu Province and Chinese Academy of Sciences QianHuHouCun 1 210014 Nanjing China; ^3^ Swiss Tropical and Public Health Institute Socinstrasse 57 4002 Basel Switzerland; ^4^ Buchmann Institute for Molecular Life Sciences (BMLS) Goethe University Frankfurt Max-von-Laue-Strasse 15 60438 Frankfurt am Main Germany

**Keywords:** biosynthesis, peptides, *Photorhabdus*, structure elucidation, total synthesis

## Abstract

A new cyclic lipopeptide, phototemtide A (**1**), was isolated from *Escherichia coli* expressing the biosynthetic gene cluster *pttABC* from *Photorhabdus temperata* Meg1. The structure of **1** was elucidated by HR‐ESI‐MS and NMR experiments. The absolute configurations of amino acids and 3‐hydroxyoctanoic acid in **1** were determined by using the advanced Marfey's method and comparison after total synthesis of **1**, respectively. Additionally, three new minor derivatives, phototemtides B–D (**2**–**4**), were identified by detailed HPLC–MS analysis. Phototemtide A (**1**) showed weak antiprotozoal activity against *Plasmodium falciparum*, with an IC_50_ value of 9.8 μm. The biosynthesis of phototemtides A–D (**1**–**4**) was also proposed.

## Graphical abstract

**Cyclic lipopeptides (CLPs)** are important pharmaceutical lead compounds. We report a new antimalarial CLP phototemtide A with three minor derivatives, phototemtides B–D, which are generated after the silent NRPS‐encoding gene cluster *pttABC* from entomopathogenic *P. temperata* Meg1 was activated by heterologous expression in *E. coli*. The structures of these new CLPs were unequivocally identified by combining biological and chemical methods.

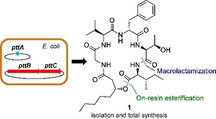

Cyclic lipopeptides (CLPs) are a class of structurally diverse natural products mainly produced by a wide variety of microorganisms, including cyanobacteria,^1^ bacteria,^2^ actinobacteria,^3^ and fungi.^4^ They are generally composed of a fatty acid tail linked to the N terminus of a short oligopeptide and the C terminus of the oligopeptide forms a lactone or lactam with a hydroxy, phenol, or amino functional group of the side chains of peptide or part of the lipid moiety.^5^ These compounds can be considered as amphiphiles due to the existence of hydrophobic lipid tails and hydrophilic amino acids or peptides, which endow them with ideal biosurfactant properties.^6^ Although the biological activity of CLPs is often reduced to these biosurfactant properties,^5^ they also displayed potent and selective antibacterial,^7^ antifungal,^8^ antiprotozoal,^9^ and cytotoxic activities.^10^ Daptomycin, isolated from *Streptomyces roseoporus*, is the first clinically used CLP antibiotic with a new structural type and unique mechanism of action.^11^ It was approved by FDA in 2003 for the nontopical treatment of complicated skin and skin structure infections caused by Gram‐positive pathogens.^11^ As one of the few newly approved antibiotics, the recent success of daptomycin highlights the evolving role of CLPs as important pharmaceutical lead compounds.^5^

The entomopathogenic bacteria of the genera *Photorhabdus* and *Xenorhabdus* that live in symbiosis with nematodes are a rich source of bioactive natural products for killing the insect, nematode development, and protecting the insect cadaver against food competitors.^12^, ^13^, ^14^, ^15^ Several CLPs, such as xenematides,^16^ xefoampeptides,^17^ chaiyaphumines,^9^ taxlllaids,^18^ and xentrivalpeptides,^19^ have been identified previously. During our further investigation on new natural products from *Photorhabdus* and *Xenorhabdus* strains, we found a new family of CLPs, named phototemtides (Scheme 1), after their biosynthetic gene cluster (BGC) *pttABC* from *Photorhabdus temperata* Meg1 was heterologously expressed in *Escherichia coli*. Here, we report the discovery, structural elucidation, biosynthesis, total synthesis, and bioactivity of the major compound phototemtide A (**1**). In addition, three minor derivatives, phototemtides B–D (**2**–**4**), were identified by detailed HPLC–MS analysis.

**Scheme 1:**
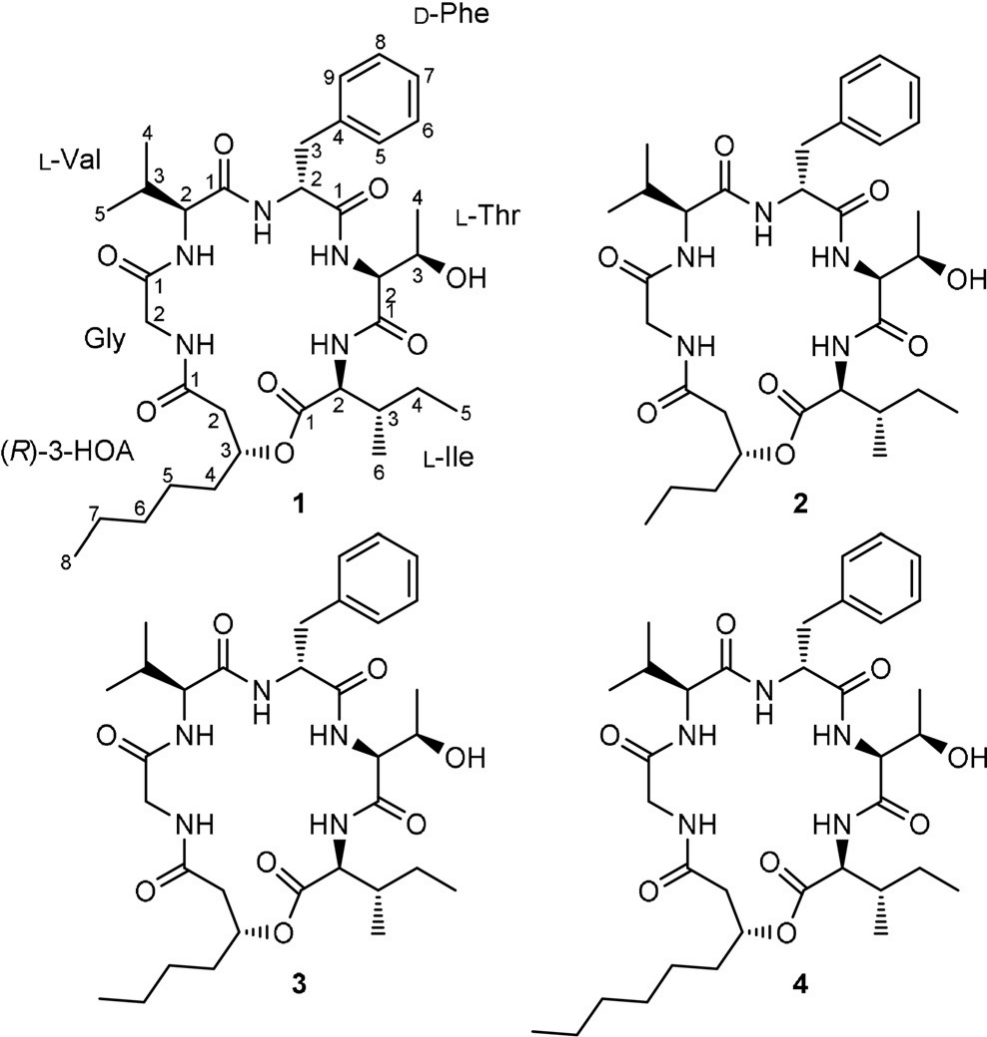
Chemical structures of phototemtides A–D (**1**–**4**).

Genome mining of *P. temperata* Meg1 (accession No. NZ_JGVH01000010) showed that the strain encodes a BGC with two nonribosomal peptide synthetases (NRPSs), termed PttB (MEG1_RS04970) and PttC (MEG1_RS04975). Detailed analysis identified that PttBC consist of five modules with overall 17 domains, including a starter condensation (C_start_) domain in the initiation module of PttB (Figure 1), which could load a fatty acid to the N terminus of oligopeptide as shown in many lipopeptides, such as anikasin,^5^ daptomycin,^20^ and taxlllaids.^18^ PttBC was thus expected to produce a lipodepsipeptide containing five amino acids. However, no such peptide could be identified in extracts of *P. temperata* Meg1 under standard laboratory conditions. Heterologous expression of an intact BGC in surrogate host has been proven a good strategy to bypass the endogenous regulatory control and activate silent pathways for the production of many interesting natural products,^21^ therefore this approach was applied to activate *pttBC* in well‐characterized *E. coli*. Notably, one MbtH‐encoding gene, termed *pttA* (*MEG1_RS04960*, 195 bp) is located 0.8 kb upstream from *pttB*. The MbtH proteins have been proposed to play an important role in stimulating adenylation reactions that are required for biosynthesis of some nonribosomal peptides.^22^, ^23^ Our effort expressing *pttBC* together with *pttA* in *E. coli* resulted in successful production of phototemtides A–D (Figure S1 in the Supporting Information). Indeed, *E. coli* was found incapable of producing phototemtides without MbtH‐encoding gene *pttA* (Figure S1).

**Figure 1:**
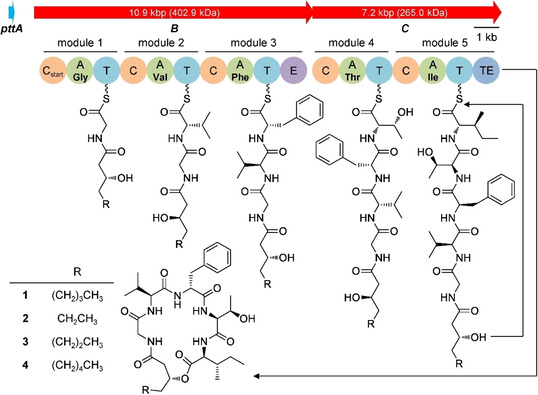
Biosynthetic gene cluster and proposed biosynthesis of phototemtides A–D (**1**–**4**). Domains: C_start_: starter condensation, C: condensation, A: adenylation, T: thiolation, E: epimerization, TE: thioesterase.

To identify the structures of **1**–**4**, the major compound phototemtide A (**1**) was isolated as a white solid (4 mg) from the XAD‐16 extracts of 4 L cultures of *E. coli* expressing *pttABC*. The molecular formula of **1** was determined to be C_34_H_53_N_5_O_8_ by its HR‐ESI‐MS data (*m*/*z* 660.3984 [*M*+H]^+^, calcd for C_34_H_54_N_5_O_8_, 660.3967; Δppm 2.5), indicating eleven degrees of unsaturation. Its structure was subsequently elucidated based on detailed 1D and 2D NMR experiments (Table 1, Figure 2). The ^1^H NMR spectrum of **1** exhibited characteristics of a typical lipopeptide, illustrating five amide NH protons (*δ*_H_=8.75, 8.06, 7.91, 7.74, 7.53 ppm), six α‐amino protons (*δ*_H_=4.50, 4.17, 4.08, 4.04, 3.94, 3.44 ppm), one ester carbinol proton (*δ*_H_=4.95 ppm), and an alkyl chain (*δ*_H_=1.60–0.80 ppm). In the ^13^C NMR spectrum, six carbonyl carbon signals (*δ*_C_=171.4, 170.9, 170.8, 170.1, 169.5, 168.4 ppm) with five nitrogen‐bearing carbon signals (*δ*_C_=57.8, 57.4, 57.1, 54.6, 42.8 ppm), and two oxygenated sp^3^ carbon signals (*δ*_C_=71.9, 66.1 ppm) were observed. In addition, one phenyl group was identified on the basis of the typical chemical shifts of *δ*_H_=7.30–7.13 ppm with total integration of five protons in the ^1^H NMR spectrum and typical chemical shifts of *δ*_C_=137.9–126.2 ppm in the ^13^C NMR spectrum. Because six carbonyl carbons and one phenyl ring accounted for ten of the eleven degrees of unsaturation, compound **1** should be a monocyclic peptide. Combining ^1^H,^1^H COSY and ^1^H,^13^C HSQC NMR data, six partial structures in **1** were constructed as glycine, valine, phenylalanine, threonine, isoleucine, and 3‐hydroxyoctanoic acid (3‐HOA). The connectivity of these building blocks was established by ^1^H,^13^C HMBC. The composition and sequence of the amino acids also perfectly match the prediction of adenylation (A) domain specificity except A5 domain predicting Val but showing Ile (Table S1); the examples of an amino acid different from that predicted in the BGC analysis are not uncommon.^24^, ^25^ Thereby, compound **1** was unequivocally elucidated to be a CLP as depicted in Scheme 1, showing fatty acid involved in a lactone ring formation, as it is rare in *Photorhabdus* and *Xenorhabdus* strains and so far has only been observed for the xefoampeptides.^17^

**Table 1:** ^1^H (500 MHz) and ^13^C (125 MHz) NMR data for **1** in [D_6_]DMSO (*δ* in ppm).

Subunit	Position	*δ*_C_, type	*δ*_H_ (*J* [Hz])		Subunit	Position	*δ*_C_, type	*δ*_H_, (*J* [Hz])
Gly	1	168.4, C			Thr	1	170.1, C	
	2a	42.8, CH_2_	3.94, overlap			2	57.4, CH	4.17, m
	2b		3.44, dd (16.4, 3.9)			3	66.1, CH	3.94, overlap
	2‐NH		7.91, t (5.2)			4	19.1, CH_3_	0.83, overlap
Val	1	171.4, C				2‐NH		7.53, d (8.8)
	2	57.8, CH	4.08, dd (11.9, 5.4)		Ile	1	170.8, C	
	3	30.0, CH	1.71, td (13.6, 6.8)			2	57.1, CH	4.04, dd (9.4, 4.2)
	4	18.7, CH_3_	0.72, d (6.7)			3	35.9, CH	1.80, m
	5	18.6, CH_3_	0.50, d (6.7)			4a	24.9, CH_2_	1.47, m
	2‐NH		7.74, d (8.8)			4b		1.18, m
Phe	1	170.9, C				5	11.1, CH_3_	0.85, overlap
	2	54.6, CH	4.50, m			6	15.3, CH_3_	0.87, overlap
	3a	35.3, CH_2_	3.02, dd (13.8, 5.4)			2‐NH		8.06, d (6.8)
	3b		2.81, dd (13.8, 10.1)		3‐HOA	1	169.5, C	
	4	137.9, C				2	40.4, CH_2_	2.38, m
	5	129.2, CH	7.28, d (7.2)			3	71.9, CH	4.95, m
	6	128.0, CH	7.23, t (7.5)			4	33.3, CH_2_	1.54, m
	7	126.2, CH	7.15, t (7.2)			5	24.5, CH_2_	1.24, m
	8	128.0, CH	7.23, t (7.5)			6	30.8, CH_2_	1.24, m
	9	129.2, CH	7.28, d (7.2)			7	22.0, CH_2_	1.24, m
	2‐NH		8.75, d (6.9)			8	13.8, CH_3_	0.85, overlap

**Figure 2:**
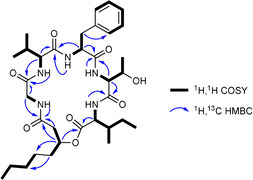
COSY and key HMBC correlations of **1**.

Analysis of MS^2^ fragmentation pattern of **1** further confirmed its structure (Figure S2). Ester bond of **1** was first cleaved to give [*M*+H]^+^ precursor ion at *m*/*z* 660 according to McLafferty rearrangement.^26^ Subsequently, the peak at *m*/*z* 642 was obtained after the loss of H_2_O from *m*/*z* 660. Further peaks at *m*/*z* 529, 428, 281, 182, and 125 indicated the consecutive losses of Ile, Thr, Phe, Val, and Gly, which is identical with amino acid composition and sequence of **1**. With these fragmentation regularities in hand, the minor derivatives **2**–**4** were identified by detailed analysis of their MS^2^ fragmentation patterns (Figure S2). They display similar constitution to **1** but are different in length of fatty acid chains (Scheme 1). As *E. coli* is not capable of producing iso‐ or anteiso‐branched fatty acids due to a missing branched‐chain keto acid dehydrogenase complex,^27^ we only expect linear fatty acids. Considering that odd‐numbered fatty acid containing natural products are uncommon in *E. coli*, a labeling experiment in lysogeny broth (LB) medium with deuterated propionic acid fed as the biosynthetic precursors of odd‐chain fatty acids^28^ was performed (Figure S3), revealing the incorporation of odd‐numbered fatty acid building block (C9) in **4**. That is to say, *E. coli* DH10B MtaA used in this study is capable of synthesizing odd‐numbered fatty acids when cultivated in LB medium, albeit in trace amounts.

To determine the absolute configurations of amino acids, compound **1** was hydrolyzed with 6 m HCl as previously described^29^ and was analyzed according to the advanced Marfey's method,^30^ resulting in the absolute configurations for Val (l), Phe (d), Thr (l), and Ile (l; Figure S4). As nonproteinogenic amino acids l‐*allo*‐Thr and l‐*allo*‐Ile are relatively uncommon in natural peptides produced by organisms,^31^, ^32^ compound **1** was more likely to feature l‐Thr and l‐Ile, which was confirmed by subsequent synthesis of **1**. The d‐Phe also matches the presence of epimerization (E) domain within module 3. The absolute configurations of amino acids in **2**–**4** were assumed to be the same as those of **1** because of their common biosynthesis origin.

However, the information regarding the stereochemistry of 3‐OH fatty acid was still missing. To address this problem, we conducted the total synthesis of two epimeric **1 a** and **1 b** containing (*R*)‐ and (*S*)‐3‐HOA moiety, respectively (Scheme 2). Due to lack of commercial availability, the synthetic work started with preparation of the chiral 3‐HOA using samarium(II) iodide (SmI_2_) mediated Reformatsky‐type reaction of (*R*)‐ or (*S*)‐4‐benzyl‐3‐bromoacetyl‐2‐oxazolidinone with *n*‐hexanal, followed by hydrolysis with lithium hydroxide and hydrogen peroxide in aqueous tetrahydrofuran.^33^ The next step was to construct the polymer‐bound tetrapeptide H_2_N‐Gly‐Val‐Phe‐Thr(*t*Bu)‐*O*‐resin by employing standard Fmoc solid‐phase peptide synthesis (SPPS) using a 2‐chlorotrityl chloride (2‐CTC) resin. Following this, the chiral lipid tails were coupled to the N terminus of the tetrapeptide chain by using 1‐[bis(dimethylamino)methylene]‐1*H*‐1,2,3‐triazolo[4,5‐*b*]pyridinium 3‐oxid hexafluorophosphate (HATU), 1‐hydroxy‐7‐azabenzotriazole (HOAt), and *N*,*N*‐diisopropylethylamine (DIPEA) in dimethylformamide (DMF). The key on‐resin esterification of 3‐HOA was then achieved with Fmoc‐Ile‐OH using a modified Yamaguchi esterification conditions with benzoyl chloride (BzCl), triethylamine (Et_3_N), 4‐(dimethylamino)pyridine (DMAP) in dichloromethane (DCM) after several attempts.^34^ Subsequently, the Fmoc‐protecting group was removed via treatment with piperidine in DMF, and the peptide was cleaved from the resin with hexafluoroisopropanol (HFIP) in DCM in order to preserve the side‐chain protecting group of Thr.^35^ Without purification, we next performed the key macrolactamization step in solution with HATU, HOAt, and DIPEA in DMF, assisted by microwave irradiation.^35^ Finally, the remaining side‐chain protecting group of peptide was removed by using trifluoroacetic acid (TFA), followed by purification using semipreparative HPLC to give **1 a** and **1 b** in 6.1 and 1.8 % overall yield based on initially actual loading of the resin, respectively. With lipid tail isomers **1 a** and **1 b** available, we assigned the absolute configuration of 3‐HOA in natural **1** to be *R* by comparison of the retention time with synthetic standards since natural **1** showed the identical retention time with synthetic **1 a** containing (*R*)‐3‐HOA moiety, while **1 b** containing (*S*)‐3‐HOA moiety eluted prior to **1**/**1 a** (Figure S5). The absolute configurations of 3‐OH fatty acids in **2**–**4** were assumed to be the same with that of **1**. Besides, the total synthesis also identified l‐Thr and l‐Ile, not l‐*allo*‐Thr and l‐*allo*‐Ile incorporated in **1**.

**Scheme 2:**
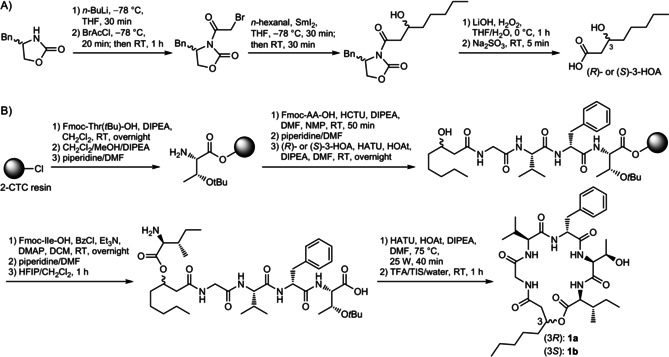
Total synthesis of epimeric **1 a** and **1 b**. A) Synthesis of chiral 3‐HOA, B) synthesis of epimeric **1 a** and **1 b**.

Bioactivity testing revealed weak antiparasitic activity of **1** against the causative agent of malaria *Plasmodium falciparum* (IC_50_=9.8 μm, Table 2), but no activity against Gram‐positive bacteria (*Micrococcus luteus*) or fungi (*Saccharomyces cerevisiae*), and no cytotoxic activity against mammalian L6 cells.

**Table 2:** Bioactivity of **1** against different protozoan parasites and mammalian L6 cells.

Species	IC_50_ [μm]	
	**1**	Control^[a]^
*Trypanosoma brucei rhodesiense*	62	0.005
*Trypanosoma cruzi*	83	2.1
*Leishmania donovani*	>100	0.73
*P. falciparum*	9.8	0.009
mammalian L6 cells	>100	0.007

[a] The control is different for each target organism: melarsoprol for *T. brucei rhodesiense*, benznidazole for *T. cruzi*, miltefosine for *L. donovani*, chloroquine for *P. falciparum*, and podophyllotoxin for L6 cells.

In summary, a new antimalarial CLP phototemtide A (**1**), with three minor derivatives, phototemtides B‐D (**2**–**4**), was identified from entomopathogenic *P. temperata* Meg1 after the silent NRPS‐encoding gene cluster *pttABC* was activated by heterologous expression in *E. coli*. The structural elucidation was accomplished by combining spectroscopic analysis and chemical methods including a total synthesis of phototemtide A. Recently, new peptide drugs have emerged due to the low toxicity, easy synthesis, rapid elimination, and less side effects,^36^ but there is currently no approved peptide alternative for the treatment of malaria. The number of marketed active ingredients is limited and most of them face challenges, such as newly observed resistance^37^, ^38^ and unpleasant side effects.^39^, ^40^ Although the bioactivity of **1** against *P. falciparum* is only weak, it might be a starting point toward a selective *P. falciparum* compound, as it shows no activity against any other tested organisms. With an efficient approach of total synthesis in hand, further investigation could focus on structure–activity relationships and subsequent in vivo experiments of this new family of CLPs. This work is also the first example of the importance of MbtH for peptide production in *Photorhabdus* and we are currently studying the role of PttA in the original producer and related strains.

## Conflict of interest

*The authors declare no conflict of interest*.

## Supporting information

As a service to our authors and readers, this journal provides supporting information supplied by the authors. Such materials are peer reviewed and may be re‐organized for online delivery, but are not copy‐edited or typeset. Technical support issues arising from supporting information (other than missing files) should be addressed to the authors.

Supplementary
