# Supplementary material for: Phototemtide A, a Cyclic Lipopeptide Heterologously Expressed from Photorhabdus temperata Meg1, Shows Selective Antiprotozoal Activity
Source: Chembiochem. 2020 Feb 4;21(9):1288–92. doi: 10.1002/cbic.201900665 (PMC7317862; doi:10.1002/cbic.201900665)
Supplement: Supplementary file 1 — Supplementary [file CBIC-21-1288-s001.pdf]

CHEMBIOCHEM

Supporting Information

Phototemtide A, a Cyclic Lipopeptide Heterologously Expressed from *Photorhabdus temperata* Meg1, Shows Selective Antiprotozoal Activity

Lei Zhao,[a, b] Tien Duy Vo,[a] Marcel Kaiser,[c] and Helge B. Bode*[a, d]

cbic_201900665_sm_miscellaneous_information.pdf

Table of Contents

Experimental Procedures **S2**
Strain Construction S2
Strain Cultivation and Culture Extraction S2
HPLC-MS Analysis S2
Compound Isolation S2
Configuration Determination of Amino Acids S3
Synthesis of Chiral 3-Hydroxyoctanoic Acids S3
Synthesis of Epimeric Peptides S5
Bioactivity Testing S7
Supplementary Tables **S8**
Table S1. A domain specificity prediction of PttABC. S8
Table S2. HRESIMS data of natural and synthetic compounds S8
Table S3. Bacterial strains used in this study. S8
Table S4. Plasmids used in this study. S8
Table S5. Primers used in this study. S9
Supplementary Figures **S10**
Figure S1. HPLC-MS analysis of XAD-16 extracts from *E. coli* expressing *pttABC* with and without arabinose induction and *E. coli* expressing *pttBC* with arabinose induction S10
Figure S2. Structure identification of **1**–**4** by detailed HPLC-MS analysis S14
Figure S3. HPLC-MS analysis of XAD-16 extracts from labeling experiments with or without [D_5] propionic acid fed to *E. coli* expressing *pttABC* in LB medium S15
Figure S4. Configuration determination of amino acids in **1** using the advanced Marfey's method S16
Figure S5. Comparison of natural **1** and synthetic **1a** and **1b** by HPLC-MS analysis S17
Figure S6. ^{1}H NMR (500 MHz, DMSO-d_6) spectrum of nature **1** S18
Figure S7. ^{13}C NMR (125 MHz, DMSO-d_6) spectrum of natural **1** S19
Figure S8. COSY (DMSO-d_6) spectrum of natural **1** S20
Figure S9. HMBC (DMSO-d_6) spectrum of natural **1** S21
Figure S10. HSQC (DMSO-d_6) spectrum of natural **1** S22
Figure S11. ^{1}H NMR (250 MHz, $CDCl_3$) spectrum of (*R*)-3-hydroxyoctanoic acid S23
Figure S12. ^{1}H NMR (250 MHz, $CDCl_3$) spectrum of (*S*)-3-hydroxyoctanoic acid. S24
Figure S13. ^{1}H NMR (500 MHz, DMSO-d_6) spectrum of synthetic **1a** S25
Figure S14. Comparison of ^{1}H NMR (500 MHz, DMSO-d_6) spectra of natural **1** (bottom) and synthetic **1a** (top) S26
Figure S15. ^{1}H NMR (500 MHz, DMSO-d_6) spectrum of synthetic **1b** S27
References **S28**

Experimental Procedures

Strain Construction

For construction of heterologous *Escherichia coli* expressing *pttABC* (*MEG1_RS04960*, *MEG1_RS04970*, and *MEG1_RS04975*) or *pttBC*, plasmids pLZ57 and pLZ58 were generated by yeast cloning[1] and Gibson cloning, respectively, and were verified by restriction enzyme digest. The correct pLZ57 was transformed into *E. coli* DH10B MtaA for coexpression with or without pLZ58. Individual colonies were analyzed by HPLC-MS for the phototemtide production.

Strain Cultivation and Culture Extraction

100 μL of overnight cultures of heterologous production *E. coli* were inoculated into 10 mL fresh liquid lysogeny broth (LB) medium supplemented with appropriate antibiotics (50 μg/mL kanamycin and spectinomycin), 0.1% L-arabinose (from a 25% stock solution), and 2% Amberlite XAD-16 resin. The cultures were grown at 30 °C and were shaken on a rotary shaker at 200 rpm. After 3 days, the cultures were harvested, and XAD-16 beads were separated and extracted with 10 mL methanol for 1 h. Subsequently, the extracts were analyzed by HPLC-MS.

HPLC-MS Analysis

HPLC-HR-ESI-MS analysis was carried out on a Dionex UltiMate 3000 system coupled to a Bruker Impact II QTOF mass spectrometer. Compounds were eluted on an ACQUITY UPLC BEH C_{18} column (130 Å, 2.1 mm × 50 mm, 1.7 μm) using a gradient from 5% to 95% acetonitrile water solution containing 0.1% formic acid at a flow rate of 0.4 mL/min for 16 min. HPLC-ESI-MS analysis was recorded on a Dionex UltiMate 3000 system coupled to a Bruker AmaZonX mass spectrometer. Compounds were eluted on an ACQUITY UPLC BEH C_{18} column (130 Å, 2.1 mm × 100 mm, 1.7 μm) using a gradient from 5% to 95% acetonitrile water solution containing 0.1% formic acid at a flow rate of 0.6 mL/min for 16 min. Positive mode with scan range from 100 to 1200 *m*/*z* was used to detect phototemtides.

Compound Isolation

To isolate phototemtide A (**1**), the XAD-16 beads from 4 L cultures of *E. coli* expressing *pttABC* were extracted with ethyl acetate. The purification of **1** was performed on an Agilent semipreparative HPLC system with an Eclipse XDB-C_{18} column (9.4 mm × 250 mm, 5 μm) using a gradient 50% to 55% acetonitrile water solution containing 0.1% formic acid for 20 min at a flow rate of 3 mL/min to afford **1** (4 mg).

Configuration Determination of Amino Acids

Amino acid configurations in **1** were determined using the advanced Marfey's method as described previously.[2,3] Approximately 1 mg of peptide was dissolved in 200 μL MeOH and was hydrolyzed with 800 μL HCl (6 M) in an Ace high-pressure tube at 110 °C for 1 h. The hydrolysate was evaporated to dryness and redissolved in 100 μL H_2O. To each a half portion (45 μL) were added 10 μL 1M $NaHCO_3$ and 80 μL 1% FDLA Nα-(5-fluoro-2,4-dinitrophenyl)-L-leucinamide or D-leucinamide (L-FDLA or D-FDLA, dissolved in acetone). The brown reaction vials were incubated in a water bath at 40 °C for 1 h. After that the reactions were cooled to room temperature, quenched with 10 μL 1 M HCl and evaporated to dryness. The residue was dissolved in 400 μL MeOH. The analysis of the L- and LD-FDLA-derivatized amino acids was carried out with HPLC-MS.

Synthesis of Chiral 3-Hydroxyoctanoic Acids

The synthesis was carried out by employing key samarium(II) iodide (SmI_2) mediated asymmetric Reformatsky-type reaction of chiral 4-benzyl-3-bromoacetyl-2-oxazolidinone with *n*-hexanal.[4]

Step a **- Preparation of chiral 4-benzyl-3-bromoacetyl-2-oxazolidinone.** In a two-neck round-bottom flask containing a magnetic stirring bar were charged (*R*)- or (*S*)-4-benzyl-2-oxazolidinone (352 mg, 3.0 mmol) and tetrahydrofuran (THF, 12 mL) under a slight pressure of nitrogen. The flask was cooled in a dry ice−isopropanol bath (−78 °C), and a *n*-hexane solution of *n*-butyllithium (*n*-BuLi, 1.6 M, 2.1 mL, 3.3 mmol) was then added using a syringe through the septum with magnetic stirring. After 30 min, bromoacetyl chloride (BrAcCl, 482 mg, 3.1 mmol) was slowly added to the mixture at the same temperature over a period of 20 min. When the addition was completed, the dry ice bath was removed, and the mixture was allowed to warm to room temperature and stirred for an additional 1 h. The reaction was quenched with saturated aqueous potassium hydrogen phosphate (K_2HPO_4, 3 mL), and the solution was then extracted with three 3 mL portions of diethyl ether. The combined extracts were dried over magnesium sulfate ($MgSO_4$), and the solvent was removed on a rotary evaporator leaving an orange liquid. The crude product was purified by flash silica gel column chromatography to give (*R*)- or (*S*)-4-benzyl-3-bromoacetyl-2-oxazolidinone as a yellow solid (~300 mg, 33% yield).

Step b **– SmI_2 mediated Reformatsky-type reaction.** All reactions were carried out in a nitrogen atmosphere using a Schlenk tube with standard techniques for air-sensitive materials. A

mixture of *n*-hexanal (124 μL, 1.0 mmol) and (*R*)- or (*S*)-4-benzyl-3-bromoacetyl-2-oxazolidinone (299 mg, 1.0 mmol) in THF (2 mL) was slowly dropwise injected to a THF solution of SmI_2 (0.1 M, 30 mL, 3.0 mmol) at –78 °C. After stirring at −78 °C for 0.5 h, the dry ice bath was removed, and the mixture was allowed to warm to room temperature and was stirred for an additional 0.5 h. The reaction was quenched with hydrochloric acid (HCl, 0.1 M, 25 mL), and the aqueous phase was extracted with three 20 mL portions of diethyl ether. The organic layer was washed with saturated aqueous sodium thiosulfate ($Na_2S_2O_3$), and then dried over $MgSO_4$. The solvent was removed under reduced pressure, and the residue was purified by flash silica gel column chromatography to afford (*R*)-4-Benzyl-3-(*R*)-3-hydroxyoctanoyl-2-oxazolidinone or (*S*)-4-Benzyl-3-(*S*)-3-hydroxyoctanoyl-2-oxazolidinone as a white solid (~195 mg, 60% yield).

Step c **- Hydrolysis of the chiral 4-benzyl-3-3-hydroxyoctanoyl-2-oxazolidinone.** In a round-bottom flask containing a magnetic stirring bar was charged (*R*)-4-Benzyl-3-(*R*)-3-hydroxyoctanoyl-2-oxazolidinone or (*S*)-4-Benzyl-3-(*S*)-3-hydroxyoctanoyl-2-oxazolidinone (192 mg, 0.6 mmol). A solution of THF in H_2O (4:1, 3 mL) was added to the flask. The flask was cooled in an ice-water bath, and a solution of hydrogen peroxide (H_2O_2) (30% in H_2O, 273 μL, 2.4 mmol) and a solution of lithium hydroxide (LiOH, 24 mg, 1.0 mmol) in H_2O (1.2 mL) were added via syringe. The resulting mixture was stirred for 1 h at 0 °C. The septum was then removed, and a solution of sodium sulfite (Na_2SO_3, 300 mg, 2.4 mmol) in H_2O (1.8 mL) was added. The THF was removed on rotary evaporator and the remaining solution was extracted three times with dichloromethane (DCM, 3.6 mL) to remove the free oxazolidinone. The aqueous layer containing the carboxylate salt of the desired product was cooled in an ice bath and was brought to pH 1 with 6 M HCl. The aqueous solution was then extracted five times with ethyl acetate (2.4 mL). The combined ethyl acetate layers were dried over $MgSO_4$ and the solvent was removed on a rotary evaporator to give (*R*)- or (*S*)-3-hydroxyoctanoic acid (3-HOA) as a colorless oil (~85 mg, 89 % yield). Their structures were confirmed by ^{1}H NMR.

(R)-3-Hydroxyoctanoic acid: colorless oil; ^{1}H NMR (250 MHz, $CDCl_3$): δ 4.03 (dt, *J* = 8.4, 3.6 Hz, 1H), 2.59 (dd, *J* = 16.6, 3.5 Hz, 1H), 2.47 (dd, *J* = 16.6, 8.6 Hz, 1H), 1.39 (m, 8H), 0.90 (t, *J* = 6.4 Hz, 3H).

(S)-3-Hydroxyoctanoic acid: colorless oil; ^{1}H NMR (250 MHz, $CDCl_3$): δ 4.07 (m, 1H), 2.59 (dd, *J* = 16.5, 3.5 Hz, 1H), 2.48 (dd, *J* = 16.4, 8.8 Hz, 1H), 1.40 (m, 8H), 0.88 (t, *J* = 6.4 Hz, 3H).

Synthesis of Epimeric Peptides

The synthesis was accomplished by combining solid and solution phase synthesis.[5,6]

Step a **- Loading of Thr(tBu) on the 2-chlorotrityl chloride (2-CTC) resin.** A solution of Fmoc-Thr(tBu)-OH (119 mg, 0.3 mmol, 3 equiv) and *N*,*N*-diisopropylethylamine (DIPEA, 153 μL, 0.9 mmol, 9 equiv) in DCM (1.5 mL) was placed in a plastic reactor vessel filled with 2-CTC resin (63 mg, 0.1 mmol, 1.0 equiv). The resulting mixture was incubated at room temperature overnight. The remaining free binding sites were capped upon incubating twice with DCM/MeOH/DIPEA (80:15:5) for 10 min at room temperature. The resin was washed several times with dimethylformamide (DMF), MeOH, and DCM, and treated with 20% piperidine in DMF (3 × 10 min) to remove the Fmoc-protecting group. The combined filtrates were used to determine the actual loading of the resin at λ_{301} nm. Afterwards, the resin was washed with DCM and dried.

Step b **- Solid-phase peptide synthesis (SPPS).** The linear sequence was synthesized twice on the 2-CTC resin preloaded with L-Thr(tBu) on a 25 μmol scale with a Syro Wave peptide synthesizer by using standard Fmoc chemistry. The resin was placed in a plastic reactor vessel with a Teflon frit and an amount of 6 equiv of amino acid derivatives (Fmoc-D-Phe-OH, Fmoc-Val-OH, Fmoc-Gly-OH, 0.2 M) were activated in situ at room temperature with *O*-(6-Chlorobenzotriazol-1-yl)-*N*,*N*,*N'*,*N'*-tetramethyluronium hexafluorophosphate (HCTU, 0.6 M, 6 equiv) in DMF in the presence of DIPEA (2.4 M, 12 equiv) in *N*-methyl-2-pyrrolidone (NMP) for 50 min. Fmoc-protecting groups were removed with 40% piperidine in DMF for 5 min and the deprotection step was repeated for another 10 min with 20% piperidine in DMF. After each coupling and deprotection step, the resin was washed with NMP. After the addition of the final amino acid residue, the resin was washed with NMP, DMF, and DCM and dried.

Step c **- Attachment of the chiral 3-HOA on the N-terminus of polymer-bound tetrapeptide.** A solution of (*R*)- or (*S*)-3-HOA (20 mg, 0.125 mmol, 5 equiv), 1-[bis(dimethylamino)methylene]-1*H*-1,2,3-triazolo[4,5-*b*]pyridinium 3-oxid hexafluorophosphate (HATU) (48 mg, 0.125 mmol, 5 equiv), 1-hydroxy-7-azabenzotriazole (HOAt) (17 mg, 0.125 mmol, 5 equiv) and DIPEA (43 μL, 0.25 mmol, 10 equiv) in DMF (1.5 mL) was added to resin. The resulting mixture was incubated in a plastic reactor vessel equipped with a Teflon frit at room temperature overnight. The resin was washed with DMF and DCM, and was treated with 40% piperidine in DMF for 5 min and then 20% piperidine in DMF for 10 min at room temperature to remove the Fmoc-protecting group. Afterwards the resin was washed with DCM and dried.

***Step d* – Esterification and cleavage from the resin.** For the esterification of chiral 3-HOA with Fmoc-Ile-OH, resin was added with Fmoc-Ile-OH (177 mg, 0.5 mmol, 20 equiv), benzoyl chloride (BzCl, 58 μL, 0.5 mmol, 20 equiv), triethylamine (Et_3N, 140 μL, 1.0 mmol, 40 equiv), and 4-(dimethylamino)pyridine (DMAP, 1.2 mg, 0.1 mmol, 0.4 equiv) in DCM (3 mL) and was stirred at room temperature overnight. After reaction, the Fmoc-protecting group was removed by using 40% piperidine in DMF for 5 min and then 20% piperidine in DMF for 10 min at room temperature. The protected branched peptide was cleaved with 20% hexafluoroisopropanol (HFIP) in DCM at room temperature for 1 h.

***Step e* – Cyclization and full deprotection.** The peptide was cyclized in solution assisted by microwave irradiation (40 min, 25 W, 75 °C) by using HATU (19 mg, 50 μmol, 2 equiv), HOAt (6.8 mg, 50 μmol, 2 equiv), and DIPEA (17 μL, 0.1 mmol, 4 equiv) in DMF (c = 1 mM). The cyclized product was fully deprotected by incubation with 95% trifluoroacetic acid (TFA) and 2.5% triisopropylsilane (TIPS) in H_2O at room temperature for 1 h. The peptide was purified by Agilent semipreparative HPLC system to give **1a** and **1b** in (1.0 mg) 6.1% and (0.3 mg) 1.8% overall yield based on initially actual loading of the resin, respectively. Their structures were confirmed by HR-ESI-MS and ^{1}H NMR.

*Compound **1a**:* white, amorphous solid; HR-ESI-MS *m*/*z* 660.3956 $[M + H]^+$ (calcd for $C_{34}H_{54}N_5O_8$, 660.3967, Δppm 1.7); ^{1}H NMR (500 MHz, DMSO-d_6): δ 8.73 (d, *J* = 7.0 Hz, 1H), 8.05 (d, *J* = 7.0 Hz, 1H), 7.90 (m, 1H), 7.73 (d, *J* = 8.8 Hz, 1H), 7.52 (d, *J* = 8.8 Hz, 1H), 7.28 (d, *J* = 7.2 Hz, 2H), 7.23 (m, 2H), 7.15 (t, *J* = 7.2 Hz, 1H), 4.95 (m, 1H), 4.50 (m, 1H), 4.17 (dd, *J* = 8.8, 4.3 Hz, 1H), 4.08 (overlap, 1H), 4.04 (overlap, 1H), 3.94 (overlap, 2H), 3.45 (dd, *J* = 16.4, 4.3 Hz, 1H), 3.02 (dd, *J* = 13.8, 5.4 Hz, 1H), 2.81 (dd, *J* = 13.8, 10.1 Hz, 1H), 2.38 (m, 2H), 1.80 (m, 1H), 1.72 (m, 1H), 1.54 (m, 2H), 1.47 (m, 1H), 1.24 (m, 6H), 1.18 (m, 1H), 0.87 (overlap, 3H), 0.85 (overlap, 6H), 0.83 (overlap, 3H), 0.72 (d, *J* = 6.8 Hz, 3H), 0.51 (d, *J* = 6.7 Hz, 3H).

*Compound **1b**:* white, amorphous solid; HR-ESI-MS *m*/*z* 660.3957 $[M + H]^+$ (calcd for $C_{34}H_{54}N_5O_8$, 660.3967, Δppm 1.6); ^{1}H NMR (500 MHz, DMSO-d_6): δ 8.70 (d, *J* = 6.6 Hz, 1H), 7.96 (d, *J* = 8.8 Hz, 1H), 7.91 (d, *J* = 8.8 Hz, 1H), 7.82 (t, *J* = 5.6 Hz, 1H), 7.47 (d, *J* = 6.7 Hz, 1H), 7.28 (d, *J* = 7.2 Hz, 2H), 7.23 (t, *J* = 7.6 Hz, 2H), 7.16 (t, *J* = 6.7 Hz, 1H), 5.05 (d, *J* = 5.3 Hz, 1H), 4.47 (m, 1H), 4.32 (dd, *J* = 8.7, 5.3 Hz, 1H), 4.17 (dd, *J* = 8.8, 3.0 Hz, 1H), 4.10 (m, 1H), 4.02 (t, *J* = 7.2 Hz, 1H), 3.73 (dd, *J* = 16.7, 5.5 Hz, 1H), 3.66 (dd, *J* = 17.1, 5.2 Hz, 1H), 3.07 (dd, *J* = 13.9, 5.6 Hz, 1H), 2.78 (dd, *J* = 13.9, 10.0 Hz, 1H), 1.99 (m, 2H), 1.73 (m, 1H),

1.54 (m, 2H), 1.46 (m, 1H), 1.24 (m, 6H), 1.18 (m, 1H), 0.85 (overlap, 12H), 0.72 (d, J = 6.8 Hz, 3H), 0.52 (d, J = 6.7 Hz, 3H).

Bioactivity Testing

Bioactivity testing of **1** against the parasites *Trypanosoma brucei rhodesiense* STIB900, *Trypanosoma cruzi* Tulahuen C4, *Leishmania donovani* MHOM-ET-67/L82, and *Plasmodium falciparum* NF54 was carried out as described previously.[7] Cytotoxicity of **1** against rat skeletal myoblasts (L6 cells) was evaluated as described previously.[7] Bioactivity of **1** against *Micrococcus luteus* and *Saccharomyces cerevisiae* was tested using filter paper discs loaded with MeOH-dissolved compound (1 mg/mL) on the prepared plates which were incubated at 30 °C for 2 days.

Supplementary Tables

Table S1. A domain specificity prediction of PttABC.

A domain	Stachelhaus sequence	most likely amino acid predicted	amino acid detected
A1	DILQIGVIWK	Gly	Gly
A2	DAYWLGGTFK	Val	Val
A3	DAWTVAAVCK	Phe	Phe
A4	DFWNVGMVHK	Thr	Thr
A5	DAYFFGITYK	Val	Ile

Table S2. HRESIMS data of natural and synthetic compounds.

compound	sum formula	calcd. $[M + H]^+$	found $[M + H]^+$	Δppm
natural **1**	$C_{34}H_{53}N_5O_8$	660.3967	660.3984	2.5
natural **2**	$C_{32}H_{49}N_5O_8$	632.3654	632.3659	0.8
natural **3**	$C_{33}H_{51}N_5O_8$	646.3810	646.3825	2.3
natural **4**	$C_{35}H_{55}N_5O_8$	674.4123	674.4135	1.8
synthetic **1a**	$C_{34}H_{53}N_5O_8$	660.3967	660.3956	1.7
synthetic **1b**	$C_{34}H_{53}N_5O_8$	660.3967	660.3957	1.6

Table S3. Bacterial strains used in this study.

strain	genotype	reference
E. coli DH10B MtaA	F-*mcr*A, Δ(*mrr*-*hsd*RMS-*mcr*BC), Φ80*lac*ZΔM15, Δ*lac*X74, *rec*A1, *end*A1, *ara*D139, Δ(*ara leu*)7697, *gal*U, *gal*K, *rps*L, *nup*G, λ–, *ent*D::*mtaA*	[1]
P. temperata Meg1	wild type	[8]

Table S4. Plasmids used in this study.

plasmid	genotype/description	reference
pFF1	2µ ori, $G418^R$, PBAD promoter, pCOLA ori, MCS, Ypet-Flag, Km^R	[9]
pCDF-ara-tacI	3404 bp, modified from pCDF_tacI/I that contains arabinose-inducible promoter, Sm^R	Bode lab
pLZ57	25113 bp, *pttBC* from Meg1 genomic DNA assembled into pFF1, Km^R	this work
pLZ58	3560 bp, *pttA* from Meg1 genomic DNA assembled into pCDF, Sm^R	this work

Table S5. Primers used in this study.

primer	sequence (5'-3')	targeting DNA fragment	plasmid
LZ_152	TCGCAACTCTCTACTGTTTCTCCATACC CGTTTTTTTGGGCTAACAGGAGGAATTC CATGAATAAGGATGGATATT	fragment I of *pttBC* from Meg1 (6727 bp)	pLZ57
LZ_153	AGTAGAGGAGCAAGTGATGC		
LZ_154	TGTTCCATGTTGAACCGAATC	fragment II of *pttBC* from Meg1 (7557 bp)	
LZ_155	AATGCTTCATCATTGAGCGAC		
LZ_156	AGCAGAGAATGTGGCTGTTAG	fragment III of *pttBC* from Meg1 (3917 bp)	
LZ_157	TCTTCACCTTTGCTCATGAACTCGCCAG AACCAGCAGCGGAGCCAGCGGATCCGG CGCGCCTTACTCAATGCTGCTGTCTG		
LZ_148	TTGAGCAATCCATTTGAAAATGAATC	*pttA* from Meg1 (195 bp)	pLZ58
LZ_158	TCACTGCAAACTTTTTAGCCTC		
LZ_159	AGATATGAGGCTAAAAAGTTTGCAGTG ACAATTAATCATCGGCTCGTATAATG	pCDF-ara-tacI vector backbone (3423 bp)	
LZ_151	AAGAGATTCATTTTCAAATGGATTGCTC AAGGAATTCCTCCTGTTAGCCC		

Supplementary Figures

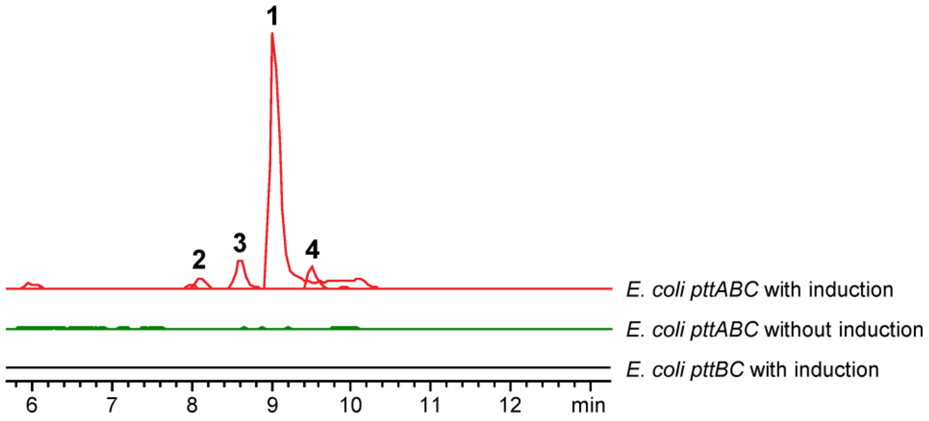

Figure S1. HPLC-MS analysis of XAD-16 extracts from *E. coli* expressing *pttABC* with and without arabinose induction and *E. coli* expressing *pttBC* with arabinose induction. Extracted ion chromatograms (EICs) for **1**–**4** are shown.

a *m/z* $[M + H]^+$ 660

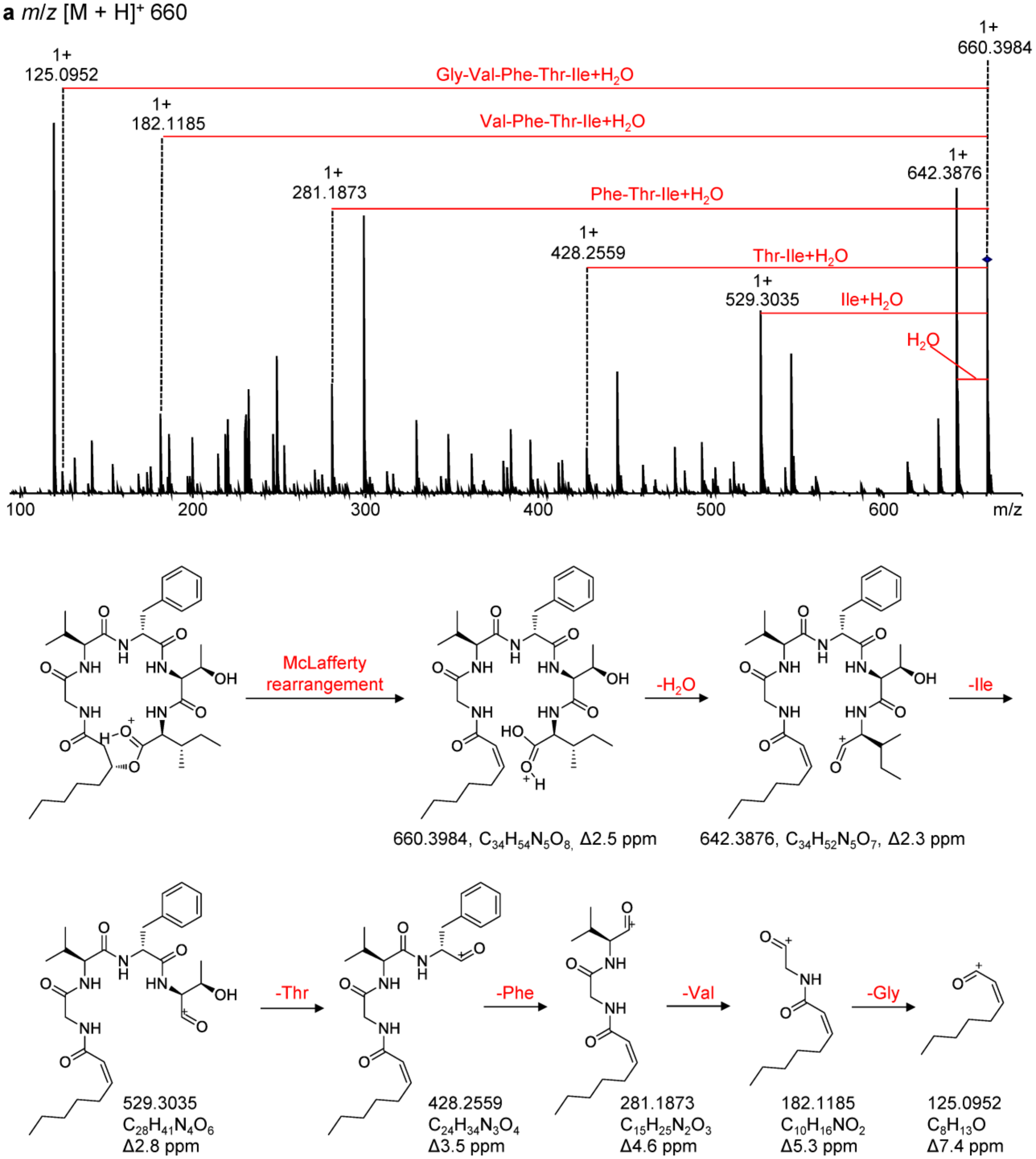

1+
660.3984
1+
125.0952
Gly-Val-Phe-Thr-Ile+H2O
1+
182.1185
Val-Phe-Thr-Ile+H2O
1+
642.3876
1+
281.1873
Phe-Thr-Ile+H2O
1+
428.2559
Thr-Ile+H2O
1+
529.3035
Ile+H2O
H2O
100
200
300
400
500
600
m/z
McLafferty rearrangement
-H2O
-Ile
660.3984, C34H54N5O8, Δ2.5 ppm
642.3876, C34H52N5O7, Δ2.3 ppm
-Thr
-Phe
-Val
-Gly
529.3035
C28H41N4O6
Δ2.8 ppm
428.2559
C24H34N3O4
Δ3.5 ppm
281.1873
C15H25N2O3
Δ4.6 ppm
182.1185
C10H16NO2
Δ5.3 ppm
125.0952
C8H13O
Δ7.4 ppm

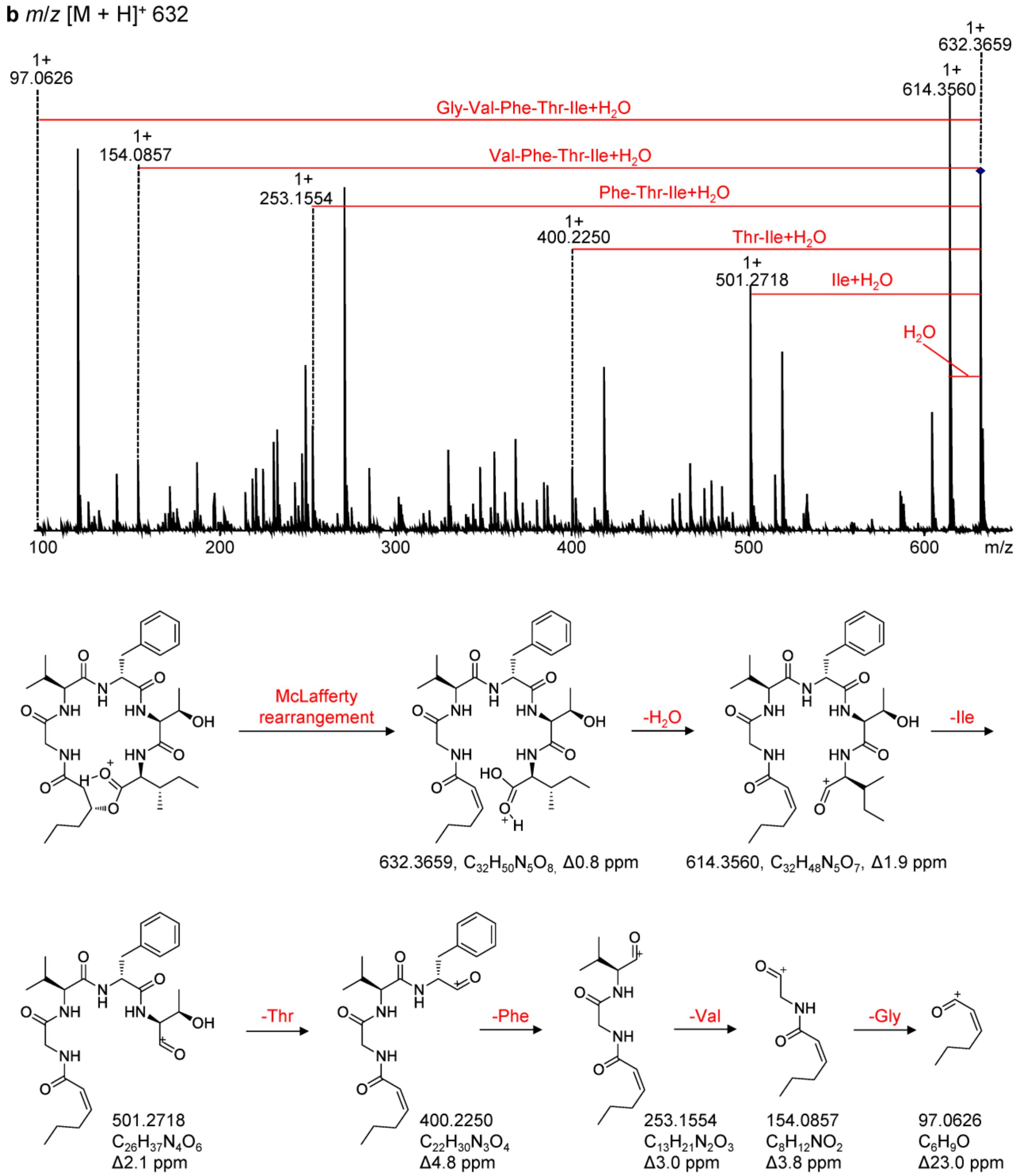

b m/z [M + H]+ 632
1+
632.3659
1+
614.3560
1+
97.0626
Gly-Val-Phe-Thr-Ile+H2O
1+
154.0857
Val-Phe-Thr-Ile+H2O
1+
253.1554
Phe-Thr-Ile+H2O
1+
400.2250
Thr-Ile+H2O
1+
501.2718
Ile+H2O
H2O
100
200
300
400
500
600
m/z
McLafferty rearrangement
-H2O
-Ile
632.3659, C32H50N5O8, Δ0.8 ppm
614.3560, C32H48N5O7, Δ1.9 ppm
-Thr
-Phe
-Val
-Gly
501.2718
C26H37N4O6
Δ2.1 ppm
400.2250
C22H30N3O4
Δ4.8 ppm
253.1554
C13H21N2O3
Δ3.0 ppm
154.0857
C8H12NO2
Δ3.8 ppm
97.0626
C6H9O
Δ23.0 ppm

c *m/z* $[M + H]^+$ 646

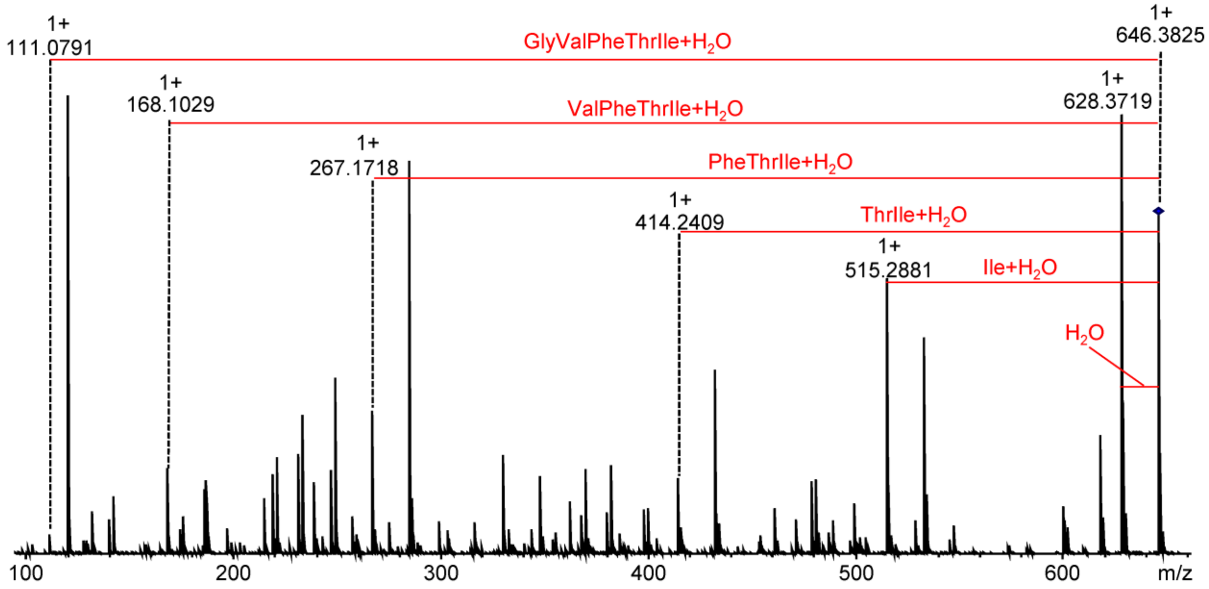

1+
111.0791
1+
646.3825
GlyValPheThrIle+H2O
1+
168.1029
ValPheThrIle+H2O
1+
628.3719
1+
267.1718
PheThrIle+H2O
1+
414.2409
ThrIle+H2O
1+
515.2881
Ile+H2O
H2O
100
200
300
400
500
600
m/z

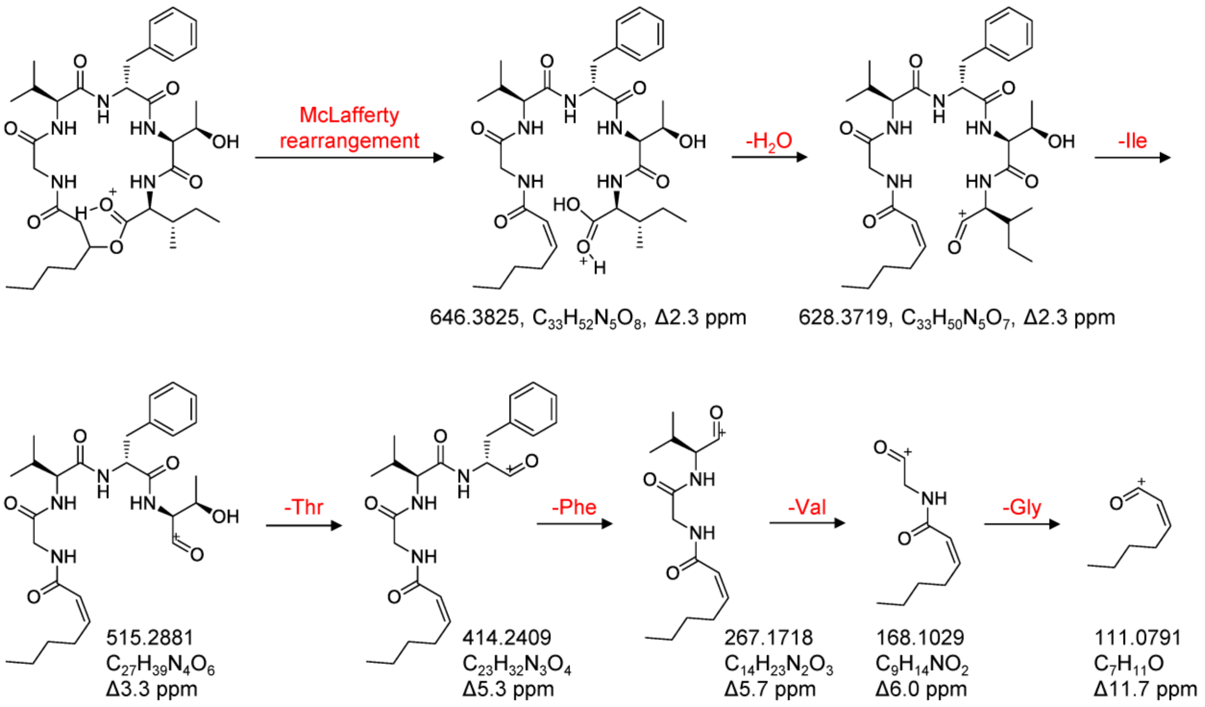

McLafferty rearrangement
-H2O
-Ile
646.3825, C33H52N5O8, Δ2.3 ppm
628.3719, C33H50N5O7, Δ2.3 ppm
-Thr
-Phe
-Val
-Gly
515.2881
C27H39N4O6
Δ3.3 ppm
414.2409
C23H32N3O4
Δ5.3 ppm
267.1718
C14H23N2O3
Δ5.7 ppm
168.1029
C9H14NO2
Δ6.0 ppm
111.0791
C7H11O
Δ11.7 ppm

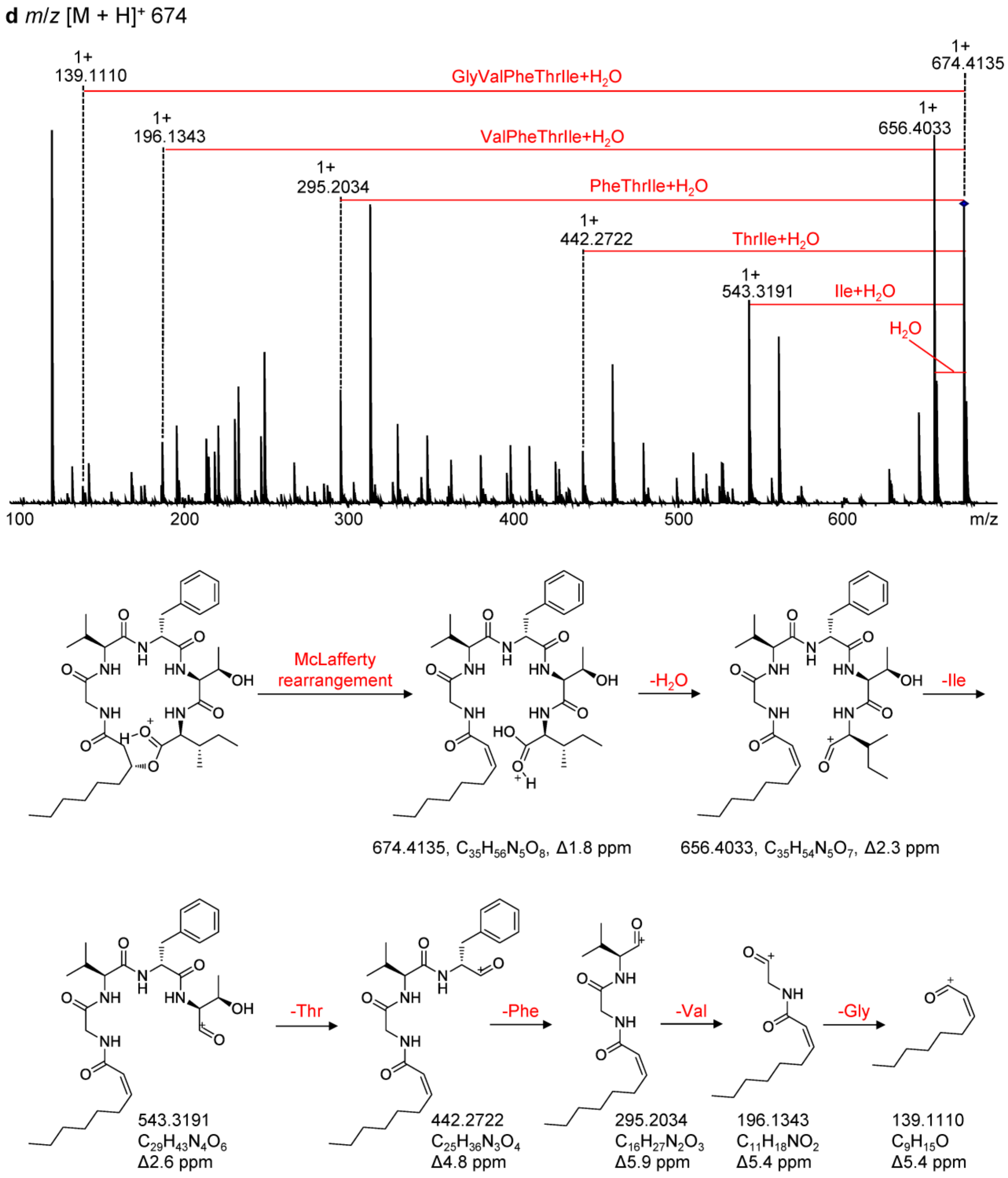

Figure S2. Structure identification of **1**–**4** by detailed HPLC-MS analysis. MS^2 spectra and fragmentation pathways with proposed fragment structures for **1** (**a**), **2** (**b**), **3** (**c**), and **4** (**d**).

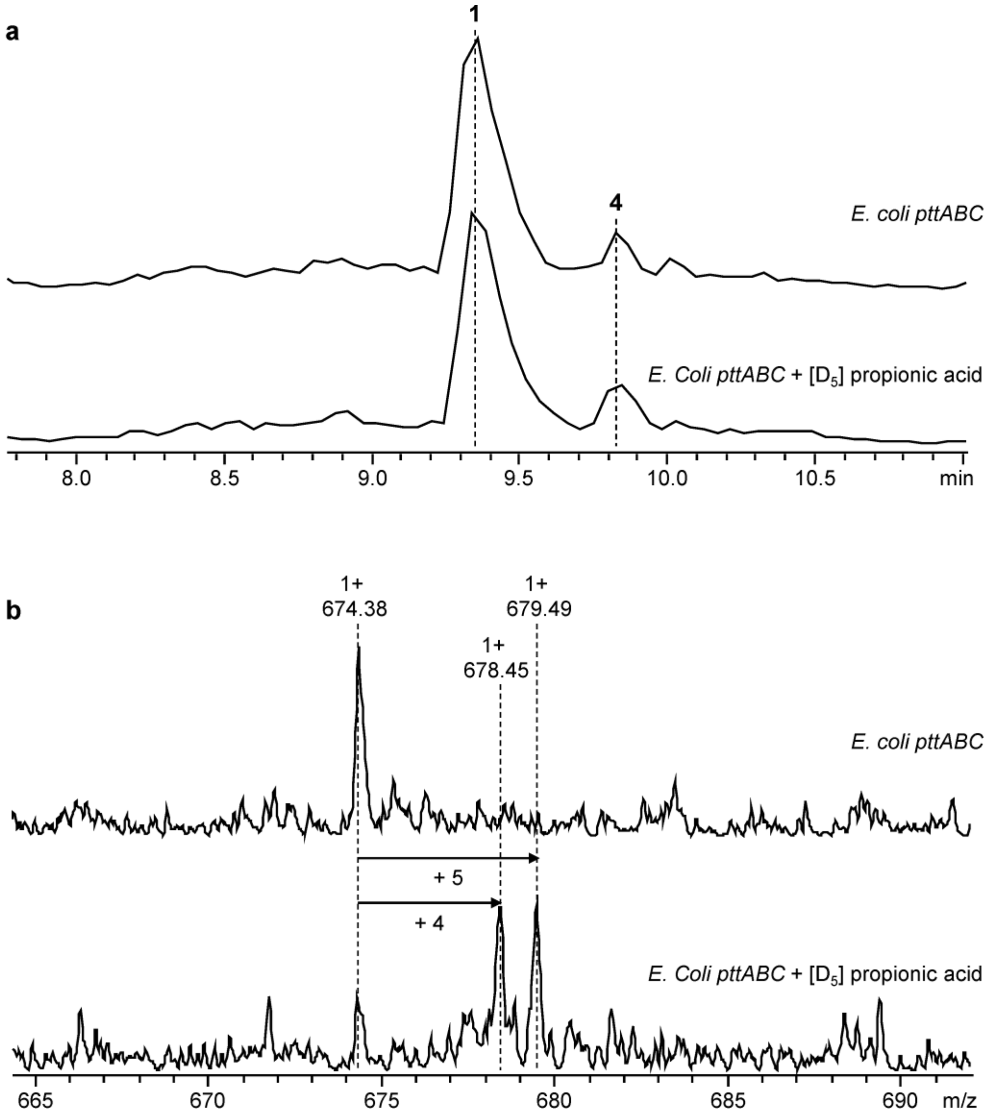

Figure S3. HPLC-MS analysis of XAD-16 extracts from labeling experiments with or without [D_5] propionic acid fed to *E. coli* expressing *pttABC* in LB medium. (**a**) Base-peak chromatograms (BPCs) for **1** and **4**; (**b**) Mass shifts for **4**.

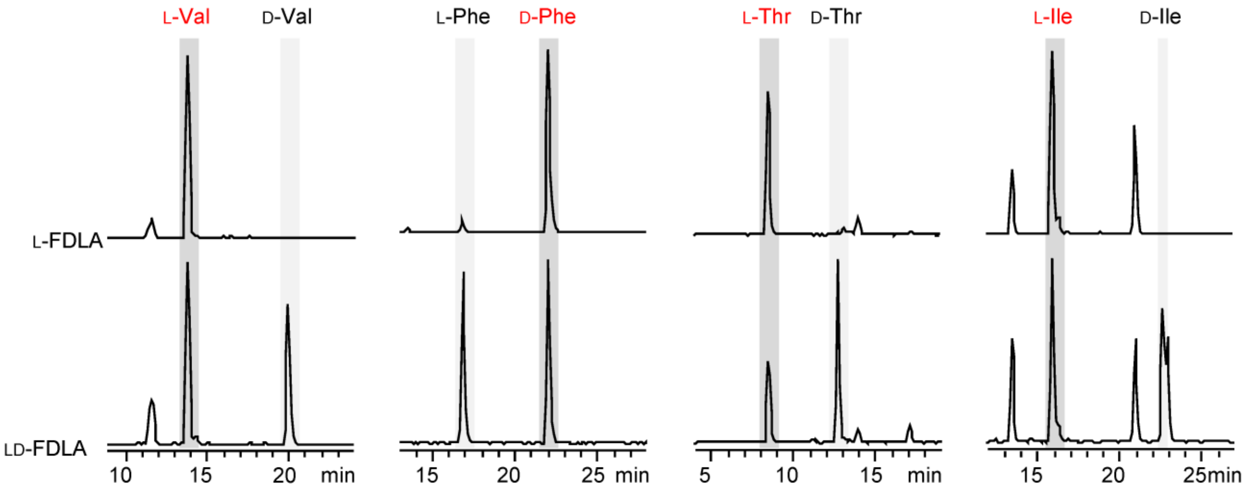

Figure S4. Configuration determination of amino acids in **1** using the advanced Marfey's method. HPLC-MS analysis of hydrolyzed **1** and derivatized with L-FDLA (upper) and LD-FDLA (lower). Depicted are EIC traces for valine (Val, *m*/*z* 412 $[M + H]^+$), phenylalanine (Phe, *m*/*z* 460 $[M + H]^+$), threonine (Thr, *m*/*z* 414 $[M + H]^+$), and isoleucine (Ile, *m*/*z* 426 $[M + H]^+$). The configurations are determined by the elution order and L-FDLA derivatized amino acids elute prior to its D-enantiomer.

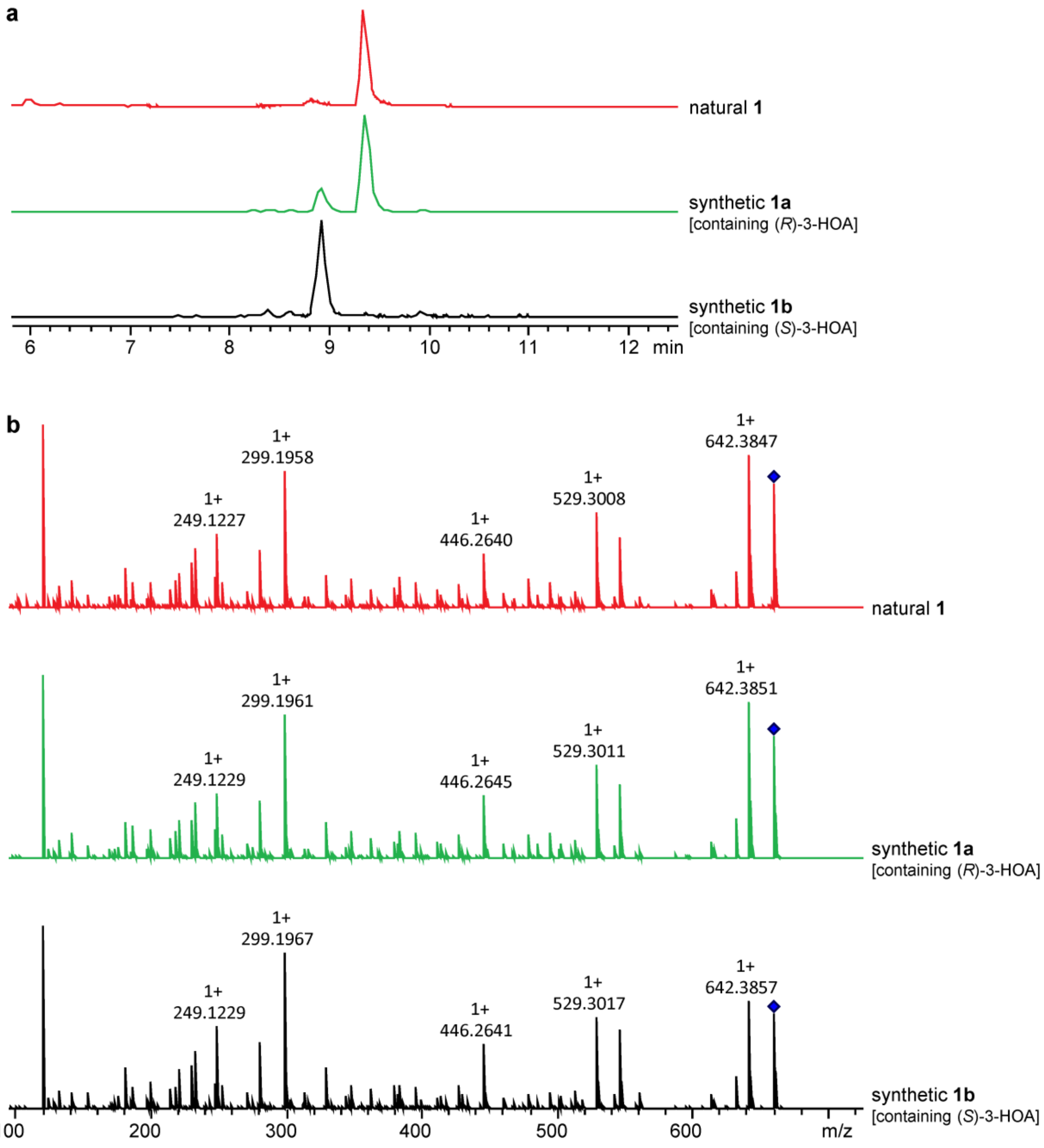

Figure S5. Comparison of natural **1** and synthetic **1a** and **1b** by HPLC-MS analysis. (**a**) EICs for natural **1** and synthetic **1a** and **1b**; (**b**) MS^2 fragmentation patterns for natural **1** and synthetic **1a** and **1b**.

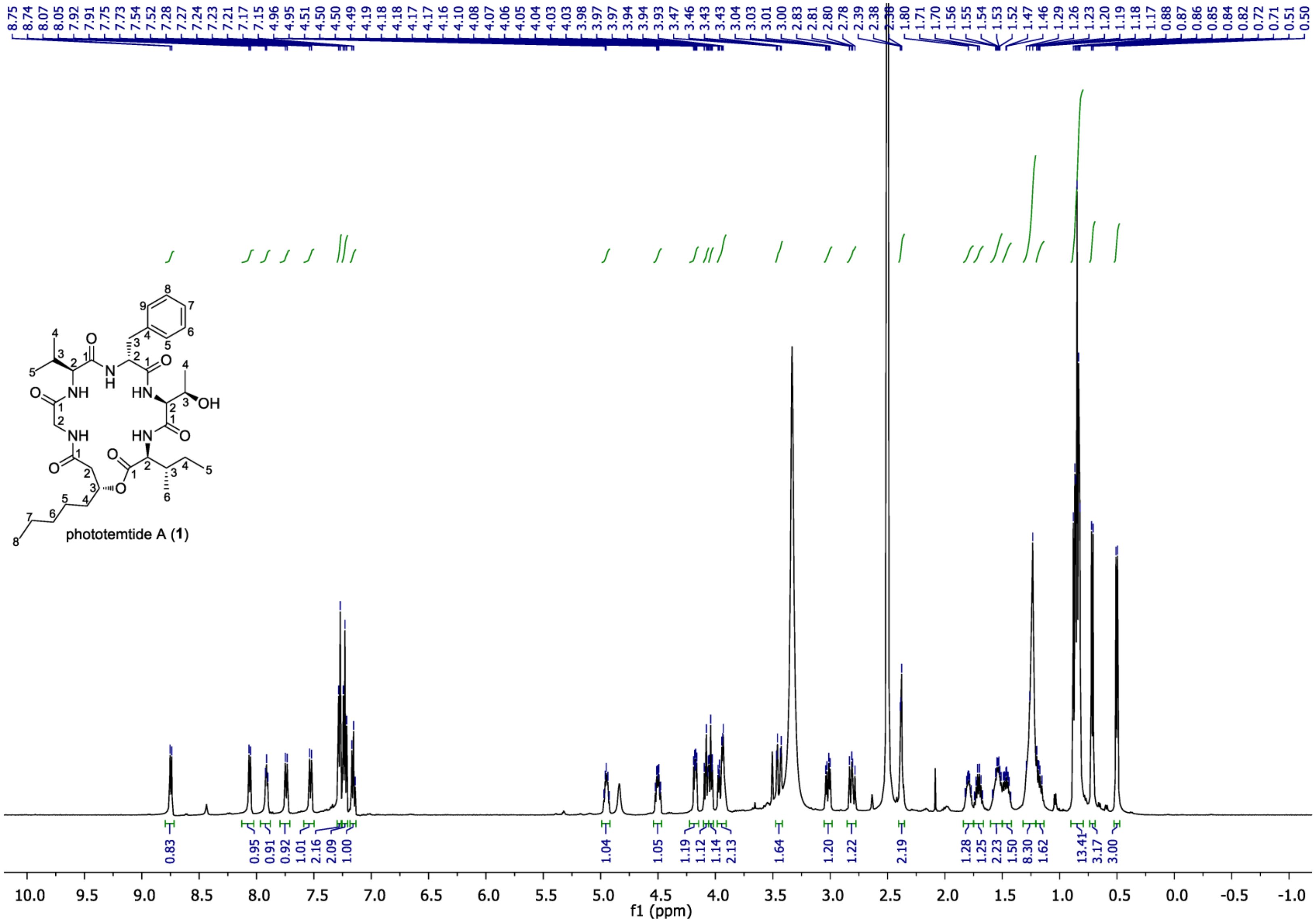

Figure S6. ^{1}H NMR (500 MHz, DMSO-d_6) spectrum of nature **1**.

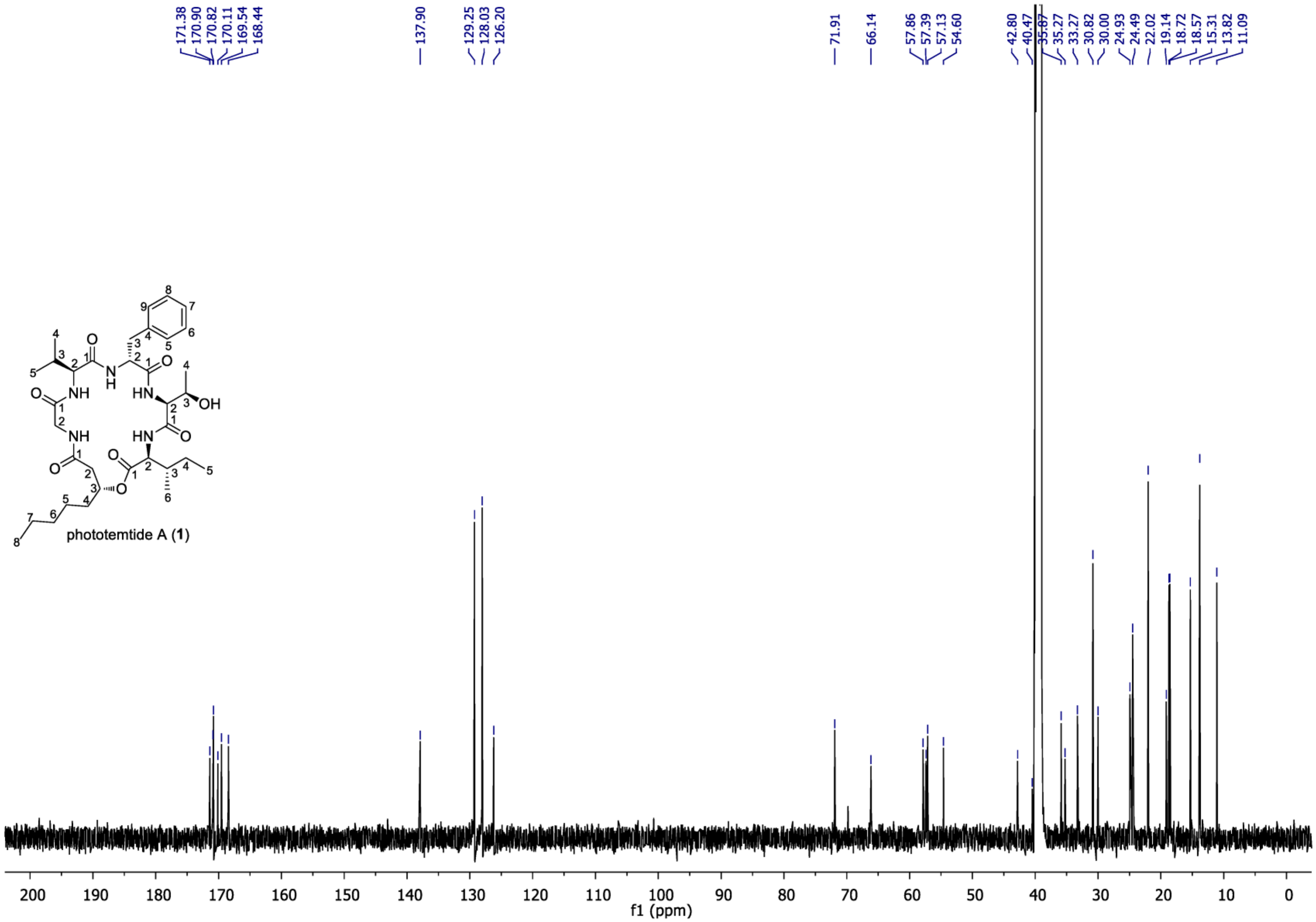

Figure S7. ^{13}C NMR (125 MHz, DMSO-d_6) spectrum of natural **1**.

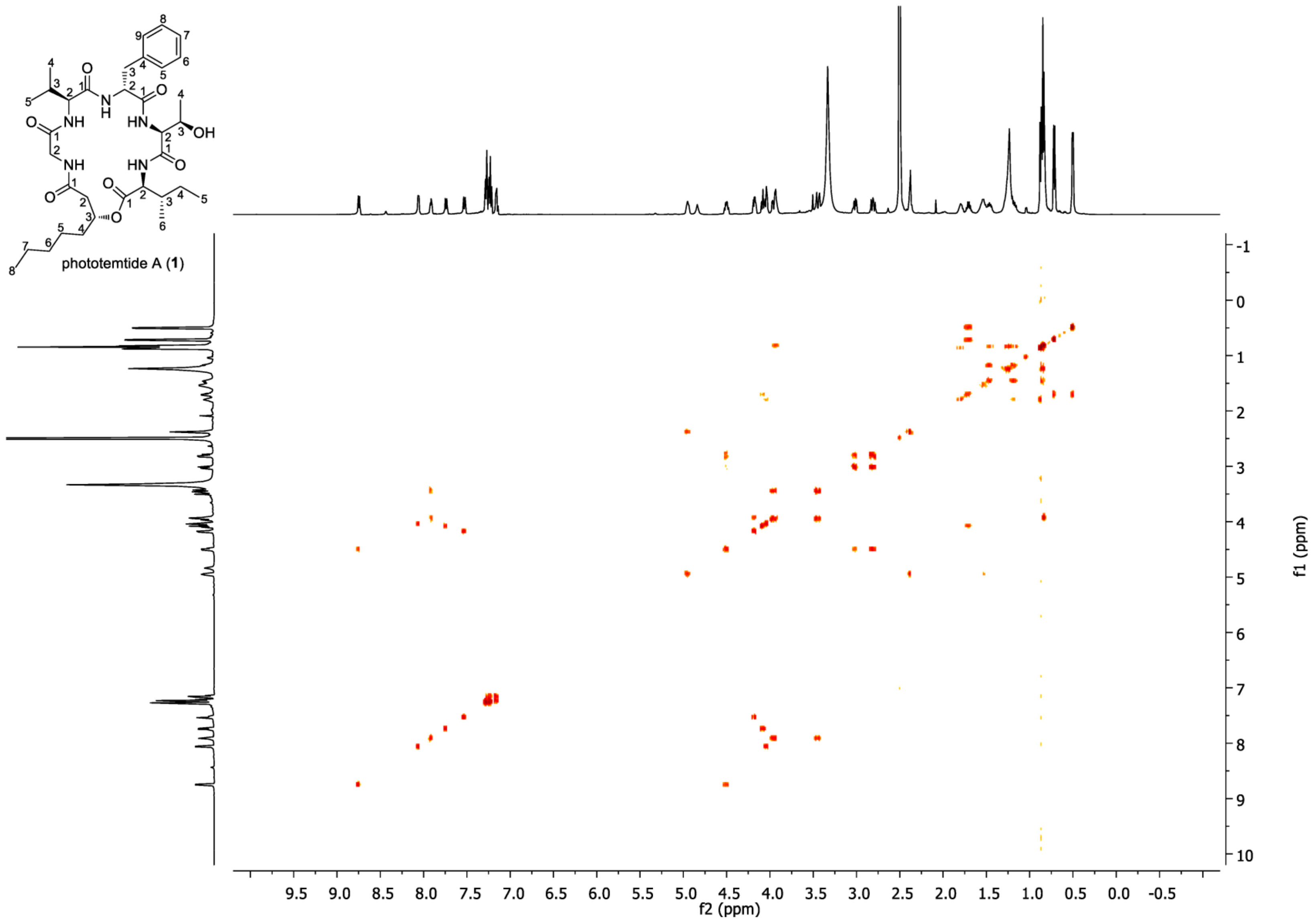

Figure S8. COSY (DMSO-d_6) spectrum of natural **1**.

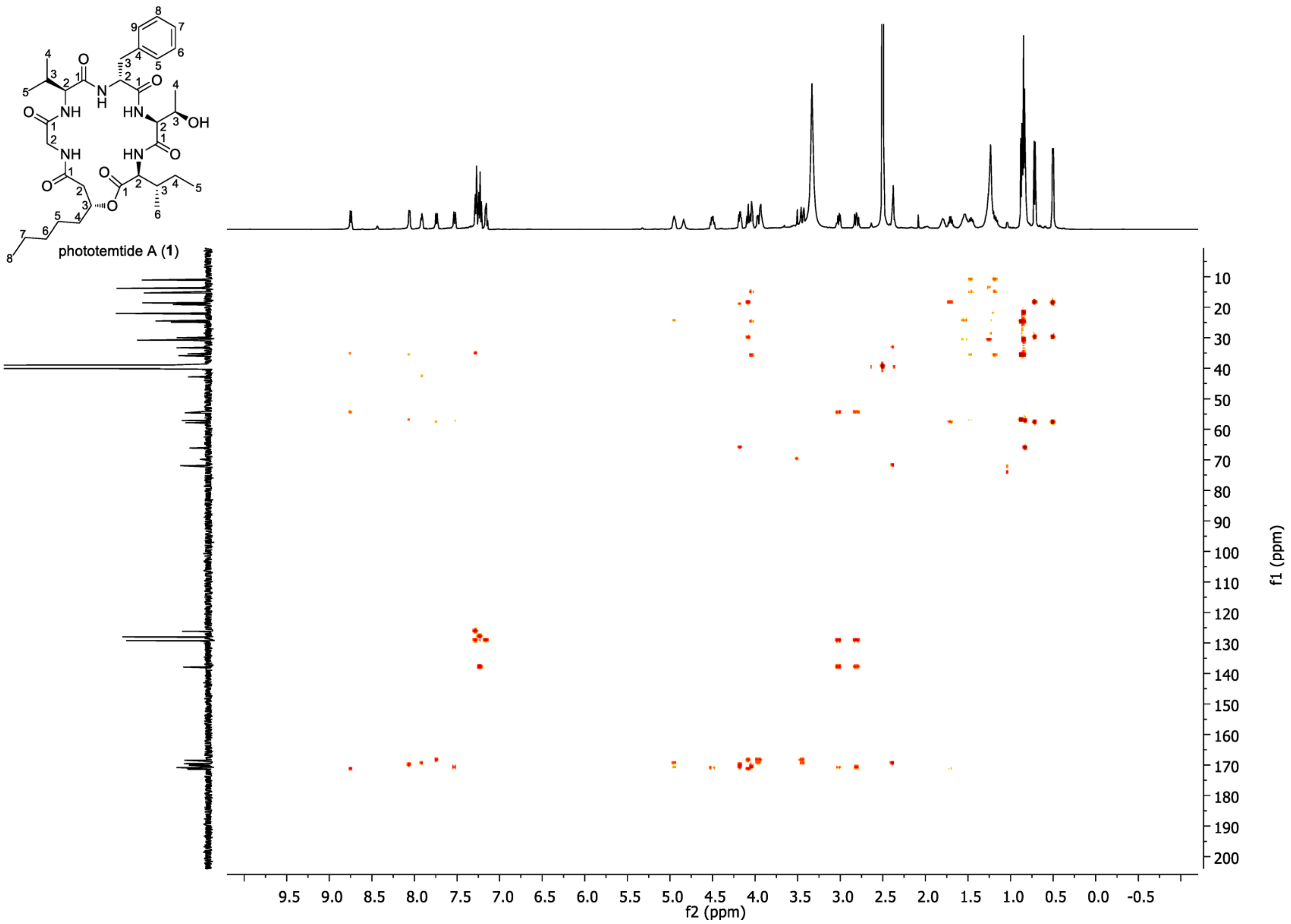

Figure S9. HMBC (DMSO-d_6) spectrum of natural **1**.

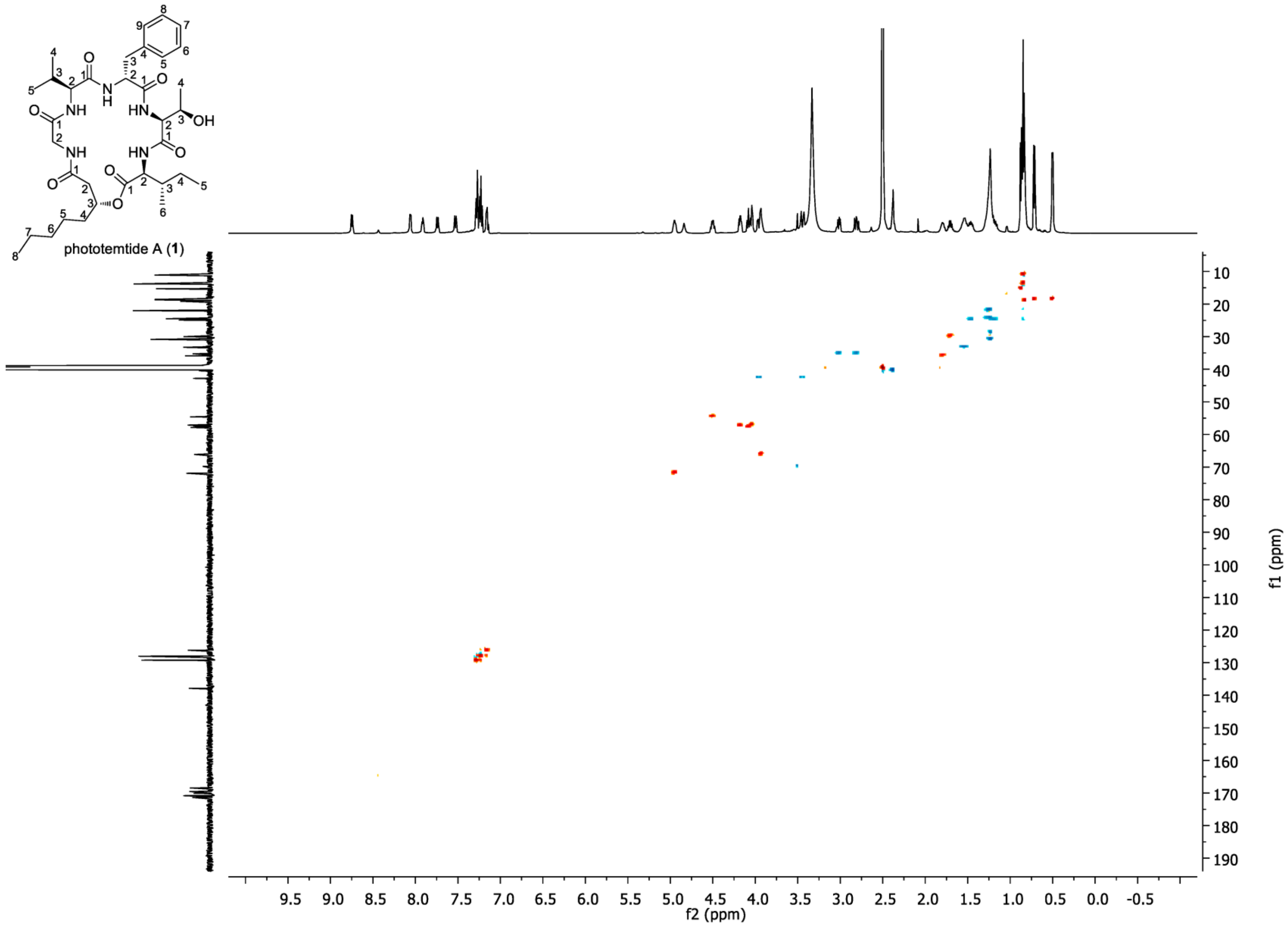

Figure S10. HSQC (DMSO-d_6) spectrum of natural **1**.

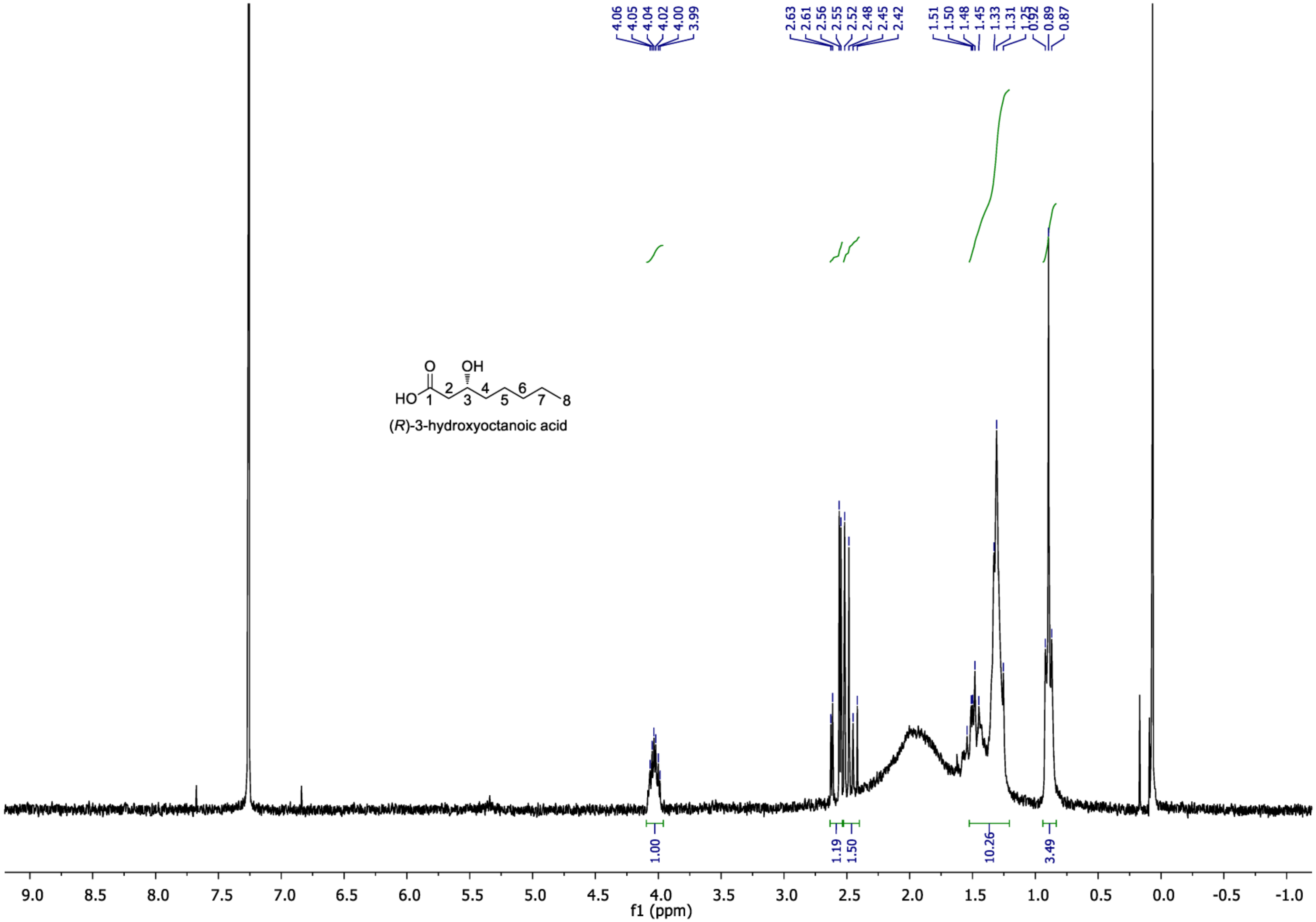

Figure S11. ^{1}H NMR (250 MHz, $CDCl_3$) spectrum of (*R*)-3-hydroxyoctanoic acid.

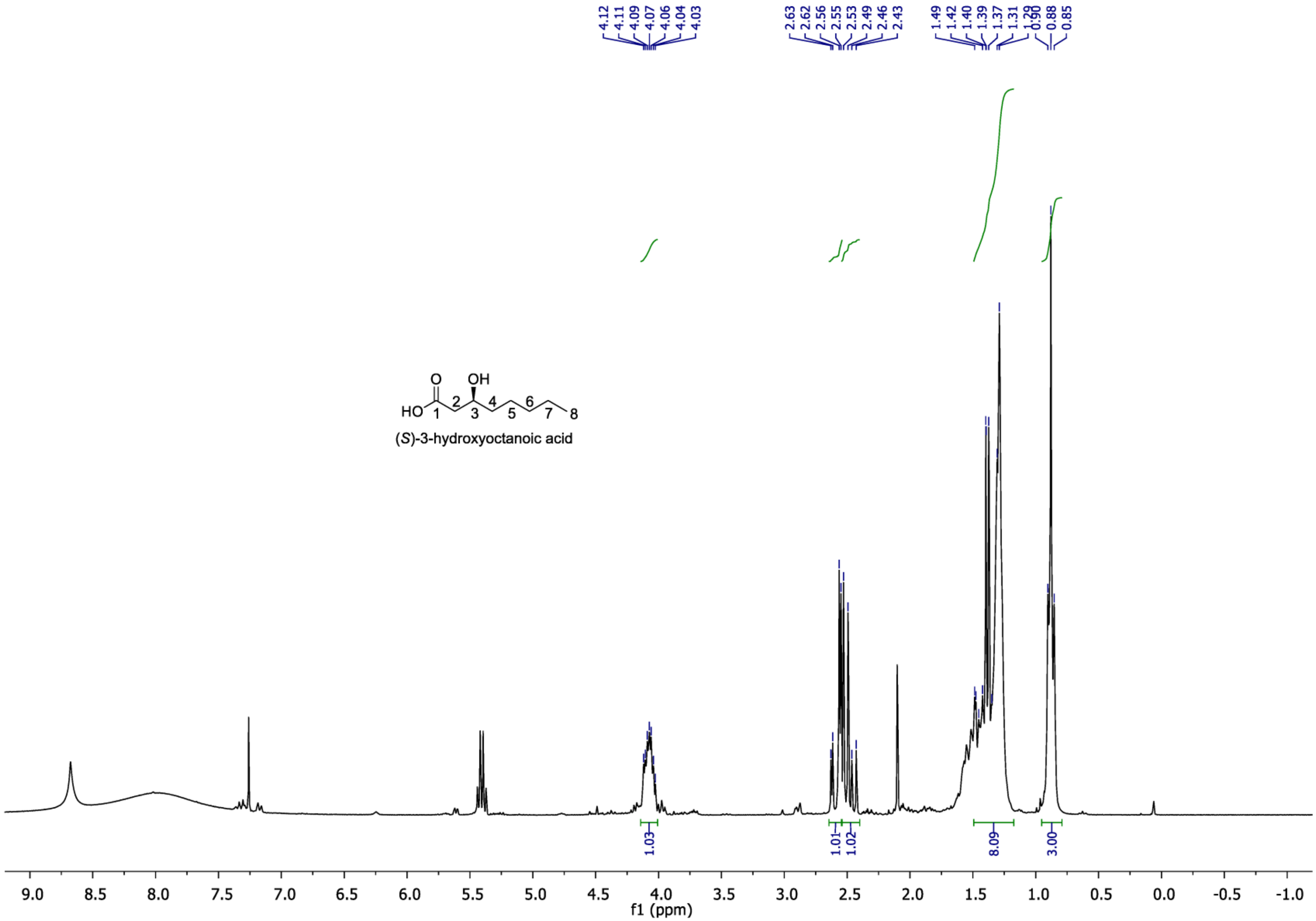

Figure S12. ^{1}H NMR (250 MHz, $CDCl_3$) spectrum of (*S*)-3-hydroxyoctanoic acid.

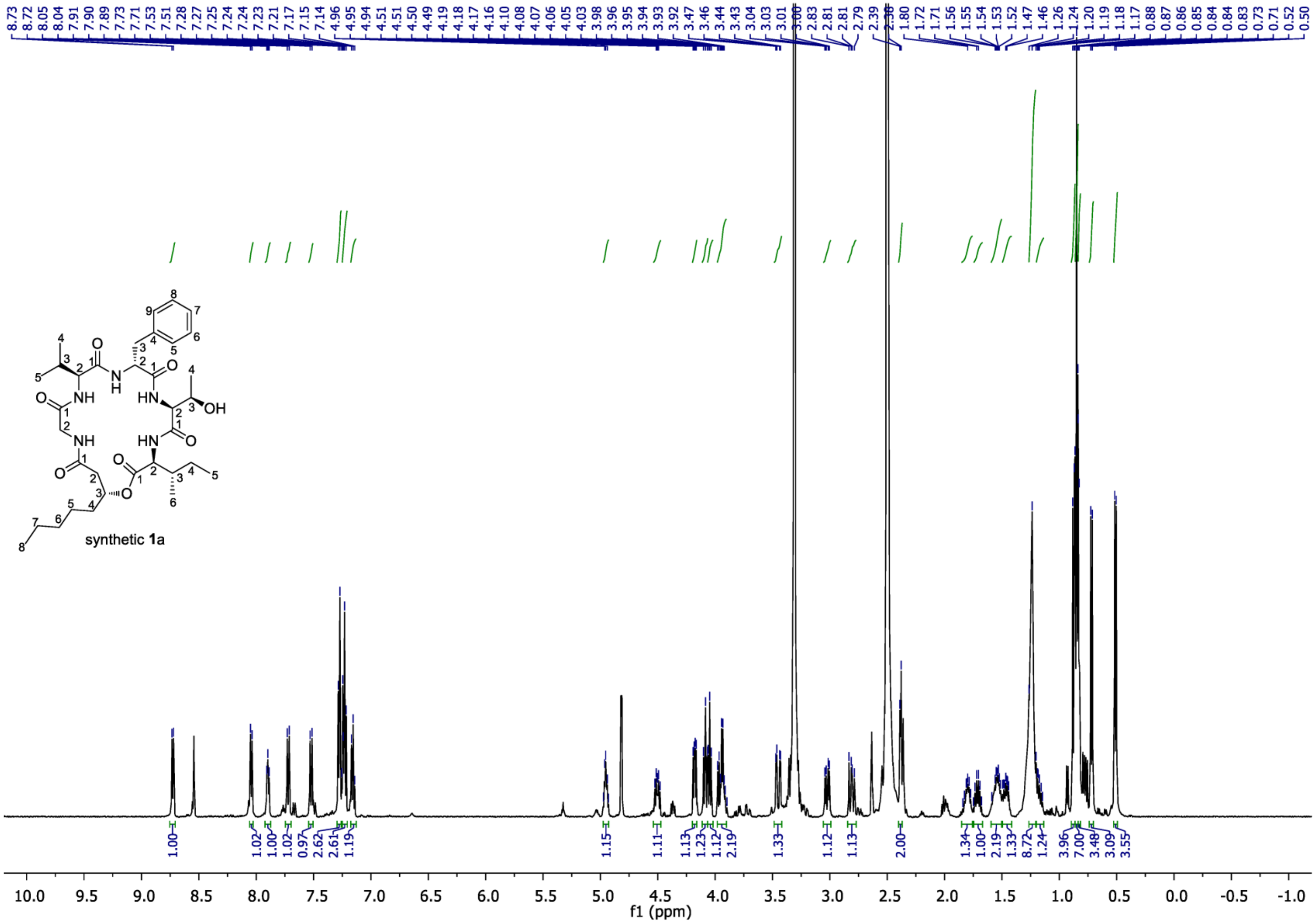

Figure S13. ^{1}H NMR (500 MHz, DMSO-d_6) spectrum of synthetic **1a**.

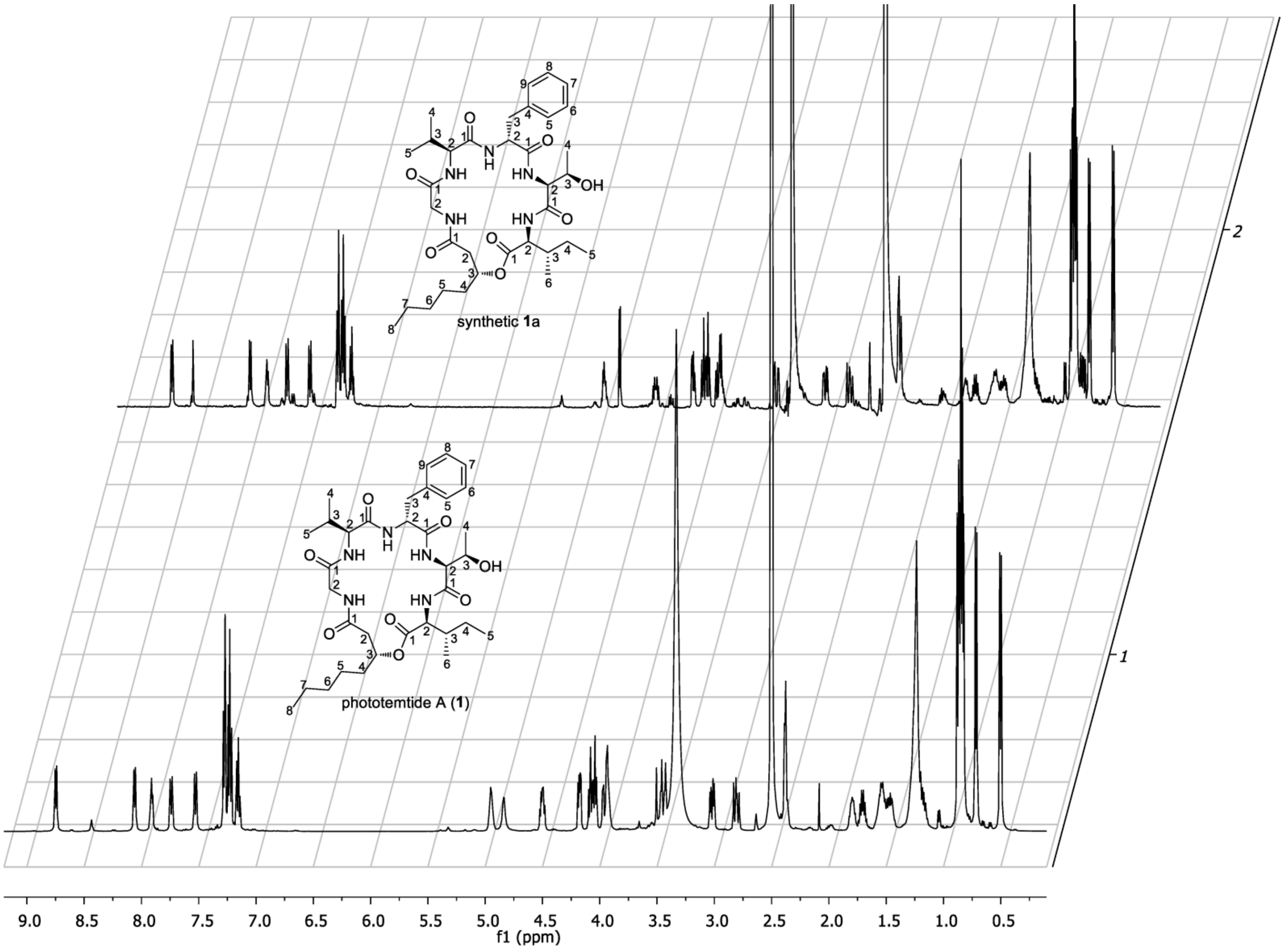

Figure S14. Comparison of ^{1}H NMR (500 MHz, DMSO-d_6) spectra of natural **1** (bottom) and synthetic **1a** (top).

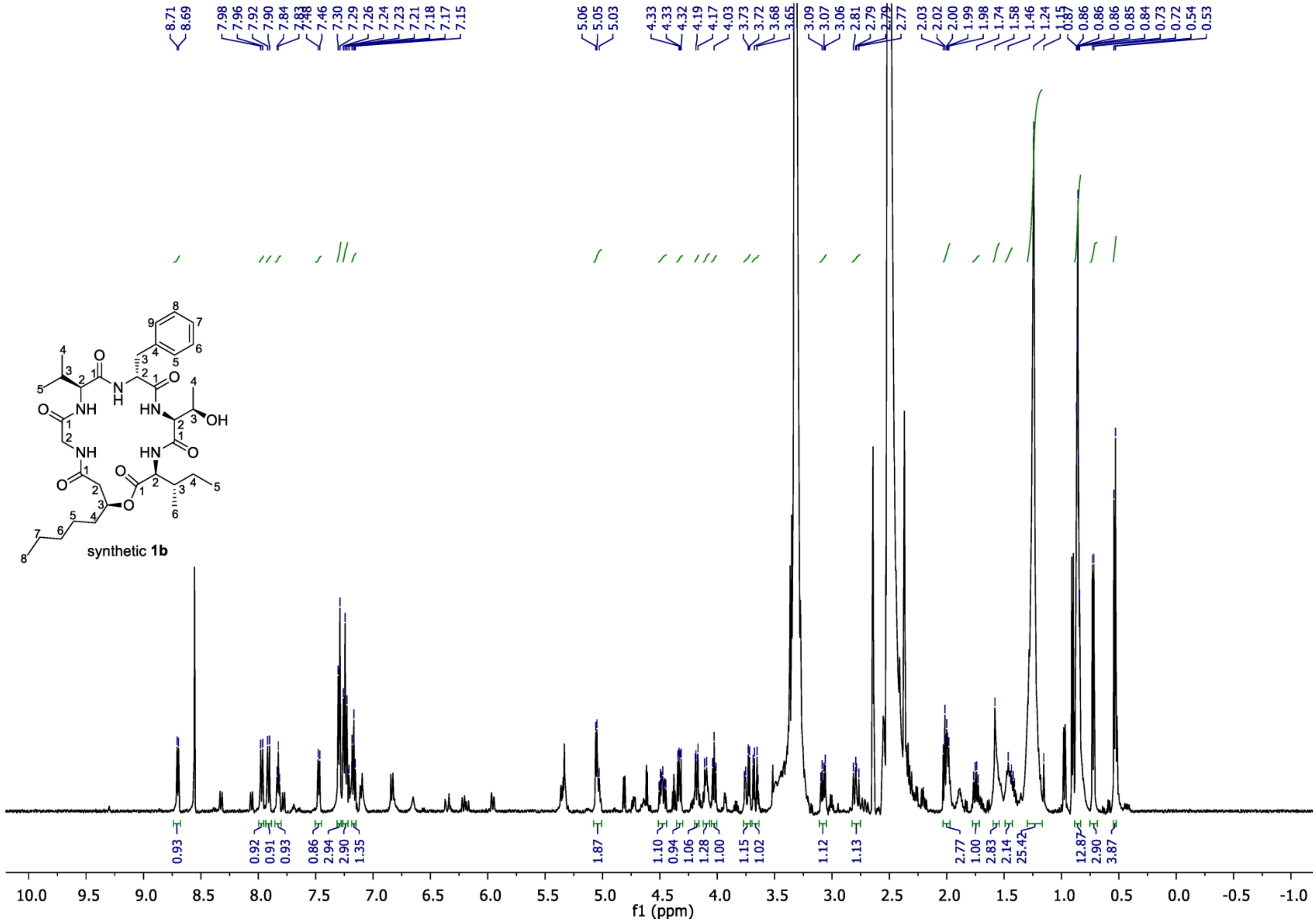

Figure S15. ^{1}H NMR (500 MHz, DMSO-d_6) spectrum of synthetic **1b**.

References

(1) Schimming, O.; Fleischhacker, F.; Nollmann, F. I.; Bode, H. B. *Chembiochem* **2014**, *15*, 1290.

(2) Grundmann, F.; Kaiser, M.; Kurz, M.; Schiell, M.; Batzer, A.; Bode, H. B. *RSC Adv.* **2013**, *3*, 22072.

(3) Fujii, K.; Ikai, Y.; Oka, H.; Suzuki, M.; Harada, K. *Anal. Chem.* **1997**, *69*, 5146.

(4) Fukuzawa, S.; Matsuzawa, H.; Yoshimitsu, S. *J. Org. Chem.* **2000**, *65*, 1702.

(5) Hung, K.; Harris, P. W. R.; Heapy, A. M.; Brimble, M. A. *Org. Biomol. Chem.* **2011**, *9*, 236.

(6) Nollmann, F. I.; Dowling, A.; Kaiser, M.; Deckmann, K.; Grösch, S.; Ffrench-Constant, R.; Bode, H. B. *Beilstein J. Org. Chem.* **2012**, *8*, 528.

(7) Orhan, I.; Şener, B.; Kaiser, M.; Brun, R.; Tasdemir, D. *Mar. Drugs* **2010**, *8*, 47.

(8) Hurst IV, S. G.; Ghazal, S.; Morris, K.; Abebe-Akele, F.; Thomas, W. K.; Badr, U. M.; Hussein, M. A.; AbouZaied, M. A.; Khalil, K. M.; Tisa, L. S. *Genome Announc.* **2014**, *2*, e01273-14.

(9) Bozhüyük, K. A. J.; Fleischhacker, F.; Linck, A.; Wesche, F.; Tietze, A.; Niesert, C.-P.; Bode, H. B. *Nat. Chem.* **2018**, *10*, 275.
